# Genomic analysis of the ecdysone steroid signal at metamorphosis onset using *ecdysoneless* and *EcRnullDrosophila melanogaster* mutants

**DOI:** 10.1007/s13258-013-0061-0

**Published:** 2013-02-05

**Authors:** Melissa B. Davis, TongRuei Li

**Affiliations:** 1 Department of Genetics, Coverdell Biomedical Research Center, University of Georgia, 500 DW Brooks Dr S 270C, Athens, GA 30602 USA; 2 Laboratory Animal Research Center, Institute of Developmental Biology and Molecular Medicine, Fudan University, 2205 Songhu Road, Shanghai, 200438 China

**Keywords:** Ecdysone, Ecdysone receptor, Genome-wide, Hormone target genes

## Abstract

Steroid hormone gene regulation is often depicted as a linear transduction of the signal, from molecule release to the gene level, by activation of a receptor protein after being bound by its steroid ligand. Such an action would require that the hormone be present and bound to the receptor in order to have target gene response. Here, we present data that presents a novel perspective of hormone gene regulation, where the hormone molecule and its receptor have exclusive target gene regulation function, in addition to the traditional direct target genes. Our study is the first genome-wide analysis of conditional mutants simultaneously modeling the steroid and steroid receptor gene expression regulation. We have integrated classical genetic mutant experiments with functional genomics techniques in the *Drosophila melanogaster* model organism, where we interrogate the 20-hydroxyecdysone signaling response at the onset of metamorphosis. Our novel catalog of ecdysone target genes illustrates the separable transcriptional responses among the hormone, the pre-hormone receptor and the post-hormone receptor. We successfully detected traditional ecdysone target genes as common targets and also identified novel sets of target genes which where exclusive to each mutant condition. Around 12 % of the genome responds to the ecdysone hormone signal at the onset of metamorphosis and over half of these are independent of the receptor. In addition, a significant portion of receptor regulated genes are differentially regulated by the receptor, depending on its ligand state. Gene ontology enrichment analyses confirm known ecdysone regulated biological functions and also validate implicated pathways that have been indirectly associated with ecdysone signaling.

**Electronic supplementary material:**

The online version of this article (doi:10.1007/s13258-013-0061-0) contains supplementary material, which is available to authorized users.

## Introduction

Steroid hormone signaling is one of the most critical mechanisms required for development and viability. Steroids control many spatiotemporal changes related to tissue function and morphology. They function in these roles for the duration of life as they are released at regimented intervals throughout the life cycle. In the clinical setting, steroid hormones have a variety of applications in correcting general developmental, reproduction and oncology; including diagnostic subtyping with hormone receptors for treatment and prognosis decisions (Doughty, 2011; Eberle et al. 2004; Eigenbrot et al. 2010; Fassnacht et al. 2011; Gangadharan et al. 2010; Hayes et al. 2011; Napieralski et al. 2010; Toft and Cryns 2011; van den Berg et al. 2011). While hormone related treatments are considered more beneficial than harmful for their specific purposes, adverse side effects on non-target (Africander et al. 2011; Buijs et al. 2008; Hospers et al. 2008; Kim and Freedland 2010) organ systems sometimes outweigh the benefit. In large part, most of the putative harms are unknown because the global impact and dynamics of target gene regulation are unknown. Accordingly, elucidating the full spectrum of steroidal gene effects is requisite to refining effective, low risk steroid treatments. While gene expression regulation is the key role of steroid signaling that has been most extensively studied to date, the dynamics of this regulation are still not fully understood.

The process of hormonal gene regulation is traditionally depicted as a linear action where the hormone is released into the circulatory system, diffuses into cells where it is bound by its specific nuclear receptor, and effectively activates the receptor. An activated receptor then binds DNA enhancer sequences in promoter regions of target genes and modulates the expression through interaction with the transcription machinery or other transcription factors, such as co-activators or co-repressors. Alternatively, the hormonal genomic regulation process could be depicted as a modular action. This model recognizes that the regulation of genes by the actual hormone molecule may occur independently of its known nuclear receptor and that similarly, some target genes are regulated by the hormone receptor independent of the hormone (Gauhar et al. 2009; Gonsalves et al. 2011). We often find in genomics investigations that target genes identified in a hormone study do not fully overlap with target genes identified in a hormone receptor investigation (Beckstead et al. 2005; Bryant et al. 1999; Giraudo et al. 2011; Terashima and Bownes 2005; Tian et al. 2010; White et al. 1999). This lack in overlap is often attributed to various factors, including desynchronized development between the sample collections, experimental artifacts as well as ‘downstream’ gene effects from loss of targeted transcription factor regulation. However, given that there is often a significant number of genes in this ‘non-overlap’ set of genes unique to each category, we purport that these recurring findings imply separable mechanisms of gene regulation between the actual hormone and its receptor(s), at least at the level of RNA expression detection. We hypothesize that in order to fully understand the complete spectrum of the hormone signal, we must be inclusive of all cascading expression changes, both overlapping with and independent of hormone receptor coupling.

Using this modular target gene overlaps perspective of hormone signal target elucidation; we now re-visit the topic of ecdysone target genes during metamorphosis. By utilizing an all-inclusive experimental design and analysis, we can fully disclose all potential hormone target genes and reveal the dynamic diversity of the signal, as both the receptor gene regulation functions and the hormone gene regulation functions are addressed independently and simultaneously. Here, we present the first steroid and steroid receptor mutant, gene expression comparison study with such a perspective.

**Fig. 1:**
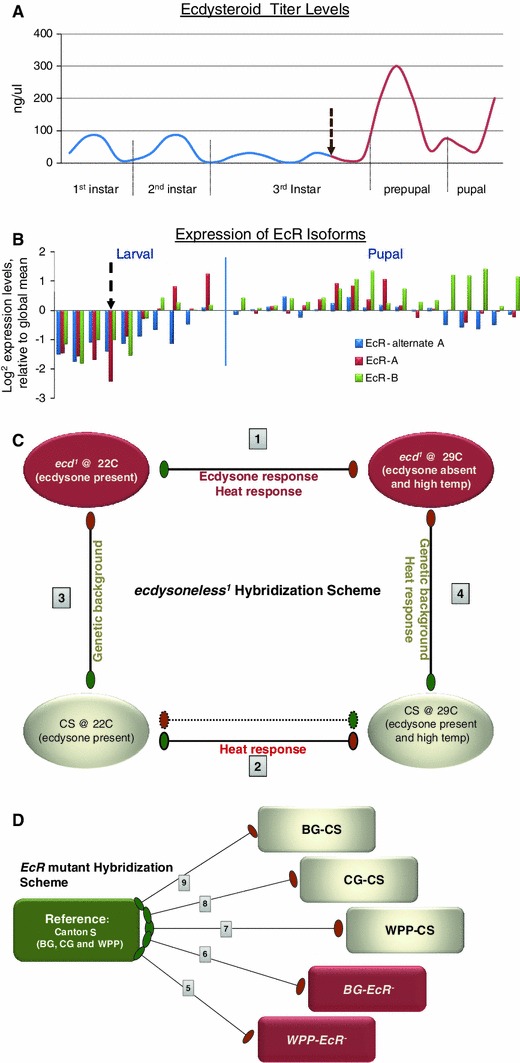
Overview of experimental design for ecdysone and Ecdysone Receptor (EcR) transcriptome responses. **a** Normal levels of 20-hydroxyecdysone pulses measured across larval and pupal stages. *Dashed arrow* indicates time point of temperature shift of *ecd* ^*1*^ mutants to restrictive temperature, removing all subsequent pulses of ecdysone. **b** Normal EcR expression during larval and pupal stages assessed by microarray analysis. Microarray probe isoform specificity is color-coded and indicated in legend. *Dashed arrows* indicate the time point where temperature rescue was ceased in *EcR*- mutants, effectively removing the expression of EcR. **c** Schematic of the *ecdysoneless (ecd* ^*1*^) hybridization experimental design, a modified round robin comparison. The samples include two wild type and two mutant (*red*) conditions. Each hybridization comparison is numbered for clarity of discussion in the text. The measured variables from each comparison is indicated and were used in our mixed model ANOVA to resolve the ecdysone-specific response, confounded by heat from the temperature shift and genomic background from the control CS samples. **d** Schematic of the *EcR*- hybridization experimental design, a global reference comparison. The reference (*green*) includes the pooled wildtype CS samples from Blue Gut (*BG*), Clear Gut (*CG*) and White Prepupae (*WPP*) stages. The experimental samples include three wildtype samples and two mutant samples (*red*), rescued up to BG and WPP stages. (Color figure online)

We have integrated classical genetics experiments with functional genomics techniques in the Drosophila model organism to elucidate the genes influenced by the 20-Hydroxyecdysone (ecdysone) steroid hormone signal. Specifically, we have resolved the target genes responding to the ecdysone signal at the specific lifecycle stage of metamorphosis onset, or pupariation (Warren et al. 2006). While this hormone is responsible for the onset of all lifecycle stages with pulses of the hormone punctuating each transition between phases of insect development (Henrich et al. 1999; Warren et al. 2006), we have chosen the pupariation pulse in order to identify both known and unknown targets during a transition of greatest diversity in morphological responses (D’Avino and Thummel 2000; Jiang et al. 2000; Kozlova and Thummel 2000; Riddiford et al. 2000). Metamorphosis onset, or the transition of Drosophila larvae into the pupal stage, is triggered by a large pulse of the ecdysone hormone (Warren et al. 2006); (Fig. 1) which is also coupled with upregulation of its receptor EcR (Fig. 1).

In our study, we undertook two independent genomic investigations, using conditional mutants, to ascertain a comprehensive set of ecdysone regulated genes. We are utilizing the extensively studied model for hormone depletion, the extensively utilized *ecdysoneless (ecd* ^*1*^ *)* temperature-sensitive mutant (Ganter et al. 2011, 2012; Gaziova et al. 2004; Henrich et al. 1993; Warren et al. 1996) and a rescue model for hormone receptor depletion, the temperature dependant transgenic *EcR*-null *(EcR*-*)* mutant developed by our group (Li and Bender 2000). We have focused the timing of removing the hormone and its receptor around the onset of metamorphosis.

Upon removing either the hormone or the receptor, the animals undergo developmental arrest at the subsequent life cycle transition stage, correlating to the next pulse of ecdysone. Before dying they usually survive for a prolonged period in the stage of development they reach upon temperature shifting (Li et al. 2001) and distinct developmental processes become inhibited or de-synchronized. From several descriptive studies of loss of ecdysone and/or the ecdysone receptor, we have an understanding of the global developmental effects from removing the signal (Henrich et al. 1993, 1999); (Ashburner 1975; Bender et al. 1997; Buszczak et al. 1999; Carney and Bender 2000; Cherbas et al. 2003; Davis et al. 2005; Li and Bender 2000) including neuronal remodeling (Schubiger et al. 1998), oogenesis (Buszczak et al. 1999; Carney and Bender 2000), cuticle production and/or shedding (Apple and Fristrom 1991; Gagou et al. 2002; Lam et al. 1999) as well as behavioral changes in feeding and wandering (Berreur et al. 1984). While we have knowledge of the gross functions that are affected due to hormone and receptor removal, it is beneficial to determine the genomic expression changes occurring, specifically at the onset of pupation when most dynamic gene expression occurs.

Using our live, conditional mutant genomics approach we identified a unique catalog of inclusive ecdysone target genes from a live animal context with all other developmental cues intact. The transcriptional profile effects we document are due to precisely removing the endogenous ecdysone signal at a very specific time point in development, with minimal artifacts from physiological manipulations.

## Materials and methods

### Sample collections from fly lines

Temperature-sensitive conditional mutants were utilized to carry out these studies. At permissive temperatures (22 °C) the *ecd* ^*1*^ mutant produces and releases the ecdysone steroid normally; however, when shifted to a restrictive temperature of 29 °C, the mutants no longer have proper release of the ecdysone hormone. Conversely, a periodic heat shock at 29 °C is required for the production of the ecdysone receptor (EcR) protein from a temperature induced mini-gene, which rescues the *EcR*- mutant. Therefore, when the mutant animals are shifted back to 22°, production of the EcR protein ceases and the animals are returned to a ‘null’ state.

#### EcR

A global reference experimental design was used for the *EcR*- comparisons. The reference was composed of an equal mixture of animals collected from stages Blue Gut (BG), Clear Gut (CG), White Pre-pupae (WPP) to WPP+10 h in 2 h intervals. The whole animal experimental samples were collected for indicated timepoints (BG, CG, WPP2a) as described previously (Li and White 2003) by removing the expression of a rescuing EcR minigene in an *EcR*- mutant background. The WPP-2a timepoint is a unique designation given to the EcR-2–3 h past the WPP stage. The animals are still alive, though developmentally halted.

#### Ecdysoneles

The *ecd* ^*1*^ mutants (Garen et al. 1977; Henrich et al. 1993) were maintained at the permissive temperature of 22 °C. Three hour egg collections were made during the peak egg lay period to synchronize colonies of animals for collections. For the mutant sampling, animals were aged at the permissive temperature to the third instar molt by measuring hours after egg lay (AEL) and then shifted to the restrictive temperature, 29 °C, just before mid-third instar wandering (Fig. 1). Because of known developmental delays in the *ecd* ^*1*^ mutant, we conducted a full life cycle staging for the mutant strain at permissive temperature. We observed and recorded, for the entire developmental time period, the actual timing of molting and pupariation. This allowed us to determine an optimal time point for shifting to the restrictive temperature in order to remove the pupariation pulse of ecdysone and prevent pupariation without interfering with the third instar molt and mid-third instar competency development. For control sampling, *ecd* ^*1*^ mutants were maintained at the permissive temperature and sampled at the White Pre-Pupa (WPP) stage. The control *ecd* ^*1*^ animals were simultaneously collected but separated from animals intended for mutant sampling during egg lay collections and maintained in parallel throughout the experiment. Once control animals at the permissive temperature reached pupariation we then took the sample of the *ecd* ^*1*^ mutants which were non-pupariating at restrictive temperatures. Additionally, *Canton S* (CS) wildtype animals were collected and treated in an identical manner to ensure minimal environmental effects.

### Microarray hybridization schemes

For the *EcR*- study we used a “reference” hybridization scheme to compare the *EcR*- stages (Fig. 1). There were five experimental conditions including three wild type conditions and two mutant conditions. The wildtype conditions are: CS at BG, CG, and WPP stages. The two mutant conditions are: BG-*EcR*- and WPP-*EcR*- which were sampled as previously described (Li and Bender 2000; Li and White 2003). Li and Bender (2000) describe the precise genotypes and crosses necessary to achieve the *EcR*- rescue. The samples obtained from each condition were hybridized to a cumulative reference sample. (See, sample collection section).

For the *ecd* ^*1*^ study we used a direct hybridization scheme for the *ecd* ^*1*^ comparisons. There were four experimental conditions: *ecd* ^*1*^ at 22° (permissive temperature (*p*- *ecd* ^*1*^)), *ecd* ^*1*^ at 29 degrees (restrictive temperature (*r*- *ecd* ^*1*^)), CS at 22° (CS-22C) and CS at 29° (CS-29C). Four direct hybridizations were done in a partial round robin arrangement where each condition was hybridized to two other conditions in a manner that allowed us to address several factors, including the removal of ecdysone, heat stress and genomic background differences (Fig. 1). In the primary and most pertinent hybridization coupling we were able to uncover genes that are differentially regulated between the mutant at permissive temperatures (22°, when ecdysone is produced) and the mutant at restrictive temperatures (29°, when ecdysone production is blocked). Inherent in this comparison was the possible contributing factor of heat stress, which would also be recovered in the differential gene list; we therefore conducted control hybridizations to test this. The second hybridization (CS-29C degrees vs. *r*-*ecd* ^*1*^) was the control done to address the heat stress possibility, removing the hormone and also removing the heat stress factor by having both strains at the same temperature. While the second hybridization removes the heat stress factor it also introduces the factor of genomic background modification differences. Ideally, the parental line of the *ecd1* mutant would have been utilized for this comparison; however, it is no longer available and therefore biases due to genomic background differences are inevitable. To address whether there is a significant contribution of genomic background modifications to alter the resulting gene expression profiles we completed a third comparison. In this comparison (CS-22C vs. *p*-*ecd* ^*1*^), the hormone is not removed, nor is there a temperature difference therefore the changes detected would solely be due to the genomic background differences. Additionally, as many genes that are heat induced are already known to also be ecdysone regulated it is not logical to simply remove genes that are potentially responsive to heat. Therefore, we decided to address the heat effect independently, leading to the fourth comparison (CS-22C vs. CS-29C) where we only introduce the heat stress as a variable in a wild type background. By overlapping the results from these four comparisons we now have the ability to discern which genes in the primary comparison are due to heat stress, and which genes in the heat stress control are due to genomic background. This approach lends the utmost confidence in characterizing the responsive genes as true ecdysone hormone targets, free of false positives due to the other variable effects of heat stress and/or genomic background modifiers.

RNA extraction and mRNA isolation and quantification were done as recommended by the manufacturer (Qiagen, mRNA isolation kits). The dye assignments were as shown in Fig. 1. Dye swapping was not necessary due to the nature of the dye assignments in the comparisons and the normalization methods used (see data analysis section below).

### Microarray design

A cDNA, PCR product platform was used in this study. The probes were produced and printed as previously described. (Li and White 2003). The array platforms utilized in this study are available at the NCBI GEO database under platform identification numbers: GPL11025, GPL11026 and GPL11027.

### Microarray data acquisition

The microarray images were scanned immediately following post-hybridization washes using an Axon Laboratories two channel scanner and the accompanying GenePix 3.0 software for raw data acquisition. These data are retrievable from the NCBI GEO under the identification number: Series GSE24486.

### Raw data analysis

#### Quality control

For each comparison, at least five hybridizations were initially done. Because these were in-house drip spotted arrays, special consideration was made for normalization, including spatial abnormalities and spreading effects. The replicates ultimately used in the analysis were chosen by pairwise correlation coefficients calculated between repeat hybridizations to reveal whether specific replicates were too divergent from others in a data set (R2, 0.8 cut off), indicating faulty hybridization or sampling artifacts. The replicates were then normalized for dye effect, background disparity and overall spatial intensity variation between repeats using a web-interface microarray analysis programs, CARMA(Rainer et al. 2006) and DNMAD (Vaquerizas et al. 2004) which incorporates a print tip loess normalization scheme via the R package program LIMMA (Smyth 2005; Smyth and Speed 2003; Wettenhall and Smyth 2004).

#### Relative expression values

**Fig. 2:**
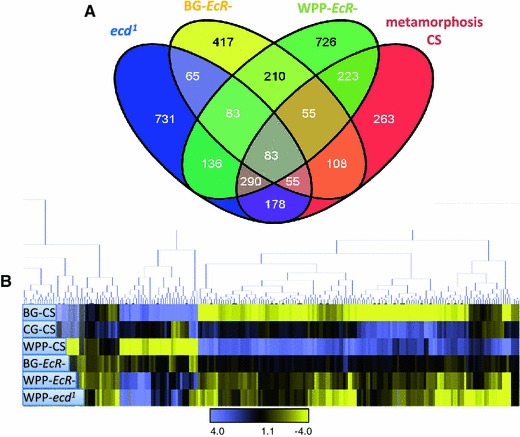
Common EcR and ecdysone sensitive target genes with identical expression responses, same polarity expression targets. **a** Hierarchical clustering revealed a subset of differentially expressed genes which share the same expression response, when either the hormone or the receptor are removed. Clear resolution of down-regulated and up-regulated nodes where observed. *Panels B* and *C*. show line graphs of gene expression changes of the common, same polarity target genes between WT to mutant categories. The down-regulated node (B) contains 204 genes that represent targets that are normally activated. The up-regulated node (C) contains 293 genes that represent targets that are normally repressed. This explicitly shows that the hormone and receptor simultaneously function as both an activation signal as well as a repression signal upon distinct subsets of target genes

For the M (regulation) and A (expression) values established between the two groups and displayed in the M–A scatter plots (Fig. 2 and supplemental Figs. 3, 4, 5, 6) the average regulation value (M) for each gene was calculated by subtracting the mean expression from the two groups (a mean M of 1 yields a twofold greater expression in group 1 compared to group 0). The average expression value (A) is simply the mean expression value for the gene. Groups for the overall ANOVA were separated into WT and mutant, including all mutant groups. Groups for the matched stage comparison were separated into lifecycle stage comparisons (“BG mutant vs. wild type at BG” and “WPP mutant vs. wildtype”).

#### Significance testing

For calculation of raw *p* values in the paired (Stage-matched) comparisons between the mutants and wild type were calculated as follows: Only genes with at least two reliable values within each group were included from the analysis. Comparisons for differential gene expression were calculated using paired moderated t-statistics provided by the Bioconductor LIMMA package to calculate the raw *p* values (Supplemental Fig. 1). To control for multiple hypothesis testing the Bonferroni adjustment method was utilized that multiplies the raw *p* value with the number of hypotheses tested in the experiment. In addition False Discovery Rates (FDR’s) were also calculated and utilized in cutoff parameters (of no more than 5 %) to establish target gene lists. Raw *p* values from the overall ANOVA analysis incorporated a mixed model which addressed the variables of removing the hormone, removing the receptor and normal hormone metamorphosis pulse and were calculated using SAS software. “Metamorphosis onset genes” were determined using a subset time course ANOVA where only the three time points of the wildtype data were analyzed for differential gene expression across the pupariation pulse. The LIMMA package was also used for this analysis.

#### Data mining

The *ecd* ^*1*^ data were further analyzed (for overlaps or relationships) by taking replicate gene averages from the significant gene list and conducting a Cluster analysis (Eisen et al. 1998) using all of the hybridization categories to determine if the significance of expression change was due to the ‘control’ external factors. Because we performed control hybridizations for the temperature and genomic background response, the clusters helped to refine the significant gene list to genes whose expression was significantly changing due to loss of ecdysone and not due to temperature and/or genomic background controlled conditions.

For the significant gene lists (wildtype, *ecd* ^*1*^, BG-*EcR*- and WPP-*EcR*-) annotation information was compiled with values of gene expression and significance scores from each comparison category (including preliminary EcR binding data) and statistically analyzed for gene network and/or biological process Gene Ontology enrichment using GenMAPP/MAPFinder software (Dahlquist et al. 2002; Doniger et al. 2003). In addition, *p* values of enrichment for Kegg pathways and Gene Ontology were calculated by using the DAVID functional annotation database(Dennis et al. 2003; da Huang et al. 2009; da Huang et al. 2008) (http://david.abcc.ncifcrf.gov/) and were treated with both the Benjamini and Bonferoni adjustment for multiple testing and the *Drosophila melanogaster* genome for background. These analyses were done both on the inclusive data set as well as on the individual conditional gene lists and subsets of overlapping genes. We used an adjusted *p* value cut off of 0.05 (Tables 1, 2, 3, 4 and 5)

**Table 1:** Gene ontology enrichment for common ecdysone signal repression targets, up-regulated by the removal of the ecdysone hormone signal

Category	Term	Count	*p* value	Bonferroni	FDR
KEGG PATHWAY	dme03050:proteasome	16	5.55E−17	1.7E−15	4.7E−14
GOTERM CC	GO:0022624 ~proteasome accessory complex	10	3.73E−13	2.6E−11	3.8E−10
GOTERM MF	GO:0070011 ~peptidase activity, acting on L-amino acid peptides	20	2.62E−09	3.5E−07	3.1E−06
GOTERM BP	GO:0030163 ~protein catabolic process	15	5.32E−10	1.9E−07	7.3E−07
GOTERM MF	GO:0004175 ~endopeptidase activity	18	1.63E−09	2.2E−07	1.9E−06
PANTHER PATHWAY	P00060:ubiquitin proteasome pathway	12	5.60E–08	7.3E − 07	3.7E−05
GOTERM BP	GO:0006508 ~proteolysis	21	9.54E−08	3.5E−05	1.3E−04
GOTERM MF	GO:0004298 ~threonine-type endopeptidase activity	6	9.96E−07	1.3E−04	1.2E−03
GOTERM BP	GO:0044265 ~cellular macromolecule catabolic process	12	1.62E−06	5.9E−04	2.2E−03
GOTERM BP	GO:0034984 ~ cellular response to DNA damage stimulus	8	7.51E−06	2.7E−03	1.0E−02
PANTHER PATHWAY	P00049:Parkinson disease	9	1.04E−04	1.3E−03	6.9E−02
PANTHER FAMILY	PTHR11599 ~ PROTEASOME SUBUNIT ALPHA/BETA	5	3.19E−05	1.8E−03	3.2E−02
PANTHER FAMILY	PTH23073 ~ 26S PROTEASE REGULATORY SUBUNIT	4	6.57E−05	3.7E−03	6.5E−02
GOTERM BP	GO:0006511 ~ ubuquitin-dependent protein catabolic process	7	1.73E−04	6.7E−02	2.4E−01
PANTHER FAMILY	PTHR10220 ~ L71-LATE PUFF ECDYSONE REGULATED PROTEIN	3	8.68E−04	4.7E−02	8.5E−01
PANTHER PATHWAY	P00013:Cell cycle	4	0.00776	9.6E−02	5.0E+00
PIR SUPERFAMILY	PIRSF001171:ATP-dependent 26 s proteinase	3	0.00141	3.7E−02	1.2E+00
COG ONTOLOGY	Posttranslational modification, protein turnover, chaperones	5	0.01116	4.4E−02	4.2E+00
GOTERM BP	GO:0048066 ~ pigmentation during development	4	0.00657	9.1E−01	8.7E+00

**Table 2:** Gene ontology enrichment for common ecdysone signal activation targets, down-regulated by removal of the ecdysone hormone signal

Category	Term	Count	*p* value	Bonferroni	FDR
GOTERM BP	GO:0019835 ~cytolysis	5	9.3E−08	1.7E−05	1.2E−04
GOTERM BP	GO:0016998!cell wall macromolecule catabolic process	5	5.5E−07	1.0E−04	6.8E−04
GOTERM BP	GO:0044036 ~cell wall macromolecule metabolic process	5	5.5E−07	1.0E−04	6.8E−04
GOTERM MF	GO:0003796 ~lysozome activity	5	4.1E−06	6.0E−04	4.9E−03
GOTERM BP	GO:0006952 ~defense response	8	3.9E−05	7.3E−03	4.9E−02
GOTERM BP	GO:0006959 ~humoral immune response	6	8.7E−05	1.6E−02	1.1E−01
GOTERM BP	GO:0019318 ~hexose metabolic process	6	2.3E−04	4.2E−02	2.8E−01
GOTERM BP	GO:0006955 ~immune response	7	2.1E−04	3.9E−02	2.6E−01
GOTERM BP	GO:0005996 ~monosaccharide metabolic process	6	3.9E−04	6.9E−02	4.8E−01
GOTERM BP	GO:0042742 ~defense response to bacterium	5	8.0E−04	1.4E−01	9.9E−01
GOTERM BP	GO:0019730 ~antimicrobial humoral response	5	7.5E−04	1.3E−01	9.3E−01
INTERPRO	IPRO15341:Glycoside hydrolase, family 38 central region	3	1.3E−03	1.5E−01	1.5E + 00
GOTERM BP	GO:0006013 ~mannose metabolic process	3	2.4E−03	3.6E−01	2.9E + 00
KEGG PATHWAY	dme00500:starch and sucrose metabolism	5	4.4E−03	1.2E−01	3.7E + 00
GOTERM MF	GO:0004559 ~alpha-mannosidase activity	3	2.1E−03	2.6E−01	2.4E + 00
GOTERM MF	GO:001592 ~mannosidase activity	3	5.1E−03	5.2E−01	5.8E + 00
GOTERM CC	GO:0000323 ~lyticvacuole	3	5.3E−03	2.5E−01	5.1E + 00
GOTERM CC	GO:0005764 ~lysosome	3	5.3E−03	2.5E−01	5.1E + 00

**Table 3:** Gene Onotology enrichment analysis for metamorphosis onset genes which overlap in both *EcR*- categories

BG-*EcR*-, WPP-*EcR*-and WT target gene ontology enrichment					
Category	Term	Count	*p* value	Bonferroni	FDR
Annotation cluster 1	Enrichment score: 2.505				
GOTERM BP	GO:0006979 ~response to oxidative stress	5	4.86E−05	1.56E−02	0.07
GOTERM BP	GO:0007568 ~aging	4	3.72E−03	7.01E−01	4.91
GOTERM BP	GO:0008340 ~ determination of adult life span	4	3.72E−03	7.01E−01	4.91
GOTERM BP	GO:0010259 ~multicellular organismal aging	4	3.72E−03	7.01E−01	4.91
Annotation cluster 2	Enrichment score: 1.437				
GOTERM BP	GO:0007559 ~histolysis	4	1.65E−03	4.15E−01	2.21
GOTERM BP	GO:0016271 ~tissue death	4	1.65E−03	4.15E−01	2.21
GOTERM BP	GO:0012501 ~programmed cell death	4	1.46E−02	9.92E−01	18.08
GOTERM BP	GO:0008219 ~cell death	4	1.70E−02	9.96E−01	20.74
GOTERM BP	GO:0016265 ~death	4	1.73E−02	9.97E−01	21.05
GOTERM BP	GO:0035071 ~salivary gland cell autophagic cell death	3	2.21E−02	9.99E−01	26.09
GOTERM BP	GO:0035070 ~salivary gland histolysis	3	2.21E−02	9.99E−01	26.09
GOTERM BP	GO:0048102 ~autophagic cell death	3	2.21E−02	9.99E−01	26.09

**Table 4:** Gene ontology enrichment analysis for metamorphosis onset genes which overlap in the *ecd* ^*1*^ category

*ecd* ^*1*^ and WT target gene ontology enrichment					
Category	Term	Count	*p* value	Bonferroni	FDR
Annotation cluster 1	Enrichment score: 6.050932369097855				
GOTERM BP	GO:0006091 ~generation of precursor metabolites and energy	23	1.58E−16	6.33E−14	0.000
GOTERM BP	GO:0006119 ~oxidative phosphorylation	15	5.20E−11	2.96E−08	0.000
GOTERM BP	GO:0055114 ~oxidation reduction	23	1.75E−07	9.98E−05	0.000
GOTERM BP	GO:0015980 ~energy derivation by oxidation of organic compounds	11	2.26E−07	1.29E−04	0.000
GOTERM BP	GO:0045333 ~cellular respiration	10	1.15E−06	6.58E−04	0.002
GOTERM BP	GO:0022900 ~electron transport chain	9	3.58E−06	2.04E−03	0.005
GOTERM BP	GO:0016310 ~phosphorylation	17	5.48E−06	3.12E−03	0.008
GOTERM BP	GO:0022904 ~respiratory electron transport chain	8	9.99E−06	5.68E−03	0.015
GOTERM BP	GO:0042775 ~mitochondrial ATP synthesis coupled electron transport	7	6.10E−05	3.42E−02	0.089
GOTERM CC	GO:0005739 ~mitochondrion	25	2.99E−09	3.17E−07	0.000
GOTERM CC	GO:0031090 ~organelle membrane	17	1.91E−05	2.02E−03	0.021
GOTERM CC	GO:0005746 ~mitochondrial respiratory chain	8	3.50E−05	3.70E−03	0.039
GOTERM MF	GO:0015078 ~hydrogen ion transmembrane transporter activity	12	1.95E−08	4.88E−06	0.000
KEGG PATHWAY	dme00190:oxidative phosphorylation	13	3.60E−05	1.58E−03	0.034
Annotation cluster 2	Enrichment score: 2.997206302737219				
GOTERM BP	GO:0022904 ~respiratory electron transport chain	8	9.99E−06	5.68E−03	0.015
GOTERM CC	GO:0005746 ~mitochondrial respiratory chain	8	3.50E−05	3.70E−03	0.039
GOTERM BP	GO:0042773 ~ATP synthesis coupled electron transport	7	8.48E−05	4.72E−02	0.124
GOTERM CC	GO:0005750 ~mitochondrial respiratory chain complex III	3	7.95E−03	5.71E−01	8.567

**Table 5:** Gene ontology enrichment analysis for inclusive common targets which show differential expression in each mutant category as well as wild type metamorphosis

All categories common targets					
Category	Term	Count	*p* value	Bonferroni	FDR
Annotation cluster 1	Enrichment score: 5.105566296986996				
GOTERM BP	GO:0019835 ~cytolysis	5	6.21E−08	2.15E−05	0.000
GOTERM BP	GO:0044036 ~cell wall macromolecule metabolic process	5	4.32E−07	1.49E−04	0.001
GOTERM MF	GO:0003796 ~lysozyme activity	5	1.87E−06	1.93E−04	0.002
GOTERM BP	GO:0019730 ~antimicrobial humoral response	6	4.29E−05	1.47E−02	0.059
GOTERM BP	GO:0009617 ~response to bacterium	6	8.17E−05	2.79E−02	0.112
GOTERM BP	GO:0016265 ~death	7	2.87E−04	9.46E−02	0.391
GOTERM BP	GO:0008219 ~cell death	7	2.78E−04	9.16E−02	0.379
Annotation cluster 2	Enrichment score: 4.074981857081641				
GOTERM BP	GO:0022609 ~multicellular organism adhesion to substrate	5	8.66E−07	3.00E−04	0.001
GOTERM BP	GO:0022608 ~multicellular organism adhesion	5	8.66E−07	3.00E−04	0.001
GOTERM BP	GO:0007594 ~puparial adhesion	5	8.66E−07	3.00E−04	0.001
PIR SUPERFAMILY	PIRSF002655:salivary glue protein	3	4.86E−04	9.20E−03	0.364
GOTERM BP	GO:0007591 ~molting cycle, chitin-based cuticle	5	1.35E−04	4.55E−02	0.184
GOTERM BP	GO:0018988 ~molting cycle, protein-based cuticle	5	1.86E−04	6.22E−02	0.253
Annotation cluster 3	Enrichment score: 2.9618972005601205				
INTERPRO	IPR008922:di-copper centre-containing	3	7.55E−04	7.49E−02	0.840
INTERPRO	IPR005203:hemocyanin, C-terminal	3	9.68E−04	9.49E−02	1.075
INTERPRO	IPR005204:hemocyanin, N-terminal	3	9.68E−04	9.49E−02	1.075
INTERPRO	IPR013788:arthropod hemocyanin/insect LSP	3	9.68E−04	9.49E−02	1.075

## Results

### Metamorphosis Onset Genes

In order to define the specific ecdysone targets genes that control the physiological changes of pupariation, we first identified differentially expressed genes of wild type (CS) animals in a small time course that includes the pre-pupal ecdysone pulse. We established a catalog of genes that are dynamically active across pupariation and we have termed these genes the “metamorphosis onset genes”. Our data indicate, with a 5 % FDR cut off, there were 1,255 genes, indicating that approximately 10 % of the genome is dynamically responsive and/or required during pupariation. This finding is in agreement with previously published data from our lab groups (White et al. 1999). A Gene Ontology enrichment analysis of this gene set yields the expected physiological pathways that are utilized during the morphological and behavioral changes that occur at this stage of development, including pupal adhesion genes, tissue morphogenesis, salivary gland cell death, apoptosis and neuronal remodeling.

### Ecdysone signal responsive genes

**Fig. 3:**
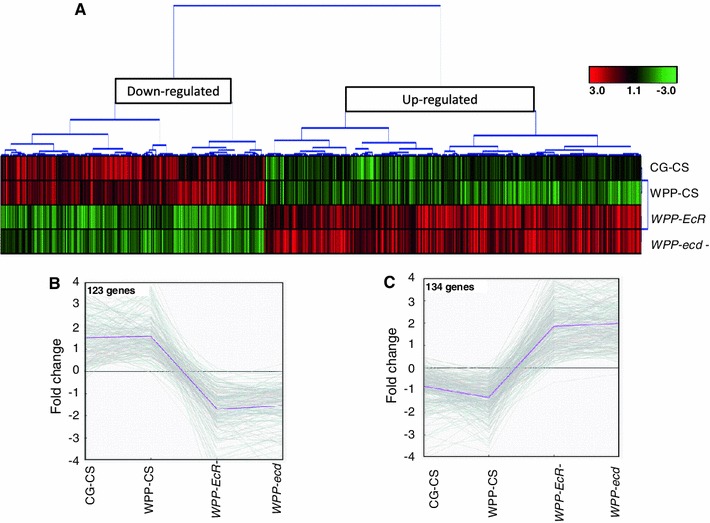
Relative overlap and clustering of significant target genes from all categories. Using our mixed model ANOVA, we see that each category has a substantial number of target genes which respond to the variables tested. Each mutant sample category is indicated and color-coded. The metamorphosis category represents a small time course of the control wild type CS line from BG to WPP+6 h. These are termed the “metamorphosis onset genes”, as they are the genes with significant changes in expression across the onset of metamorphosis. Mutant target genes which overlap with the CS metamorphosis onset genes are considered to be the valid ecdysone regulated metamorphosis onset genes. Those which are exclusive to each mutant category are most likely due to de-regulation of genes that are not normally responsive at metamorphosis, but nevertheless controlled by the hormone and the receptor

In an effort to identify novel genes which respond to the ecdysone pre-pupal pulse, we compared gene expression changes after removing either the hormone pulse or the hormone receptor at the onset of metamorphosis (Fig. 1). We utilized the *ecd* ^*1*^ and *EcR*- conditional mutant lines compared with wild type across identical developmental time points. Our initial analysis for significant genes was a mixed model ANOVA designed to identify genes that had a significant change in expression relative to the average gene expression across the onset of metamorphosis. We anticipate our list to include a subset of direct ecdysone target genes which are concordant with traditional Ashburner model early and late genes (Ashburner 1967, 1969, 1970, 1972a, b, 1974). These target genes respond to the pupariation pulse of ecdysone which triggers this transition in development and is therefore necessary for metamorphosis. As shown in the overlaps with the receptor and hormone mutants (Fig. 3a) over 60 % of the metamorphosis genes are affected by the ecdysone signal. We also identified genes that were uniquely differentially expressed in each mutant category and the overlap analysis of these genes is illustrated in Fig. 3a as well. Target gene totals for each category from our mixed model analysis are as follows: 1,621 *ecd* ^*1*^ genes, 1,076 BG-*EcR*- genes and 1,806 WPP-*EcR*-genes (gene list provided in Supplemental Table 1).

Using hierarchical cluster analysis, we see that the most significant metamorphosis onset genes (*p* value cut off of 0.001), inclusive of all categories, display a variegated expression response between mutant categories when the ecdysone signal is disrupted (Fig. 3b). Interestingly, there are nodes of genes that have opposing polarity in expression response, depending on whether the hormone or the receptor is removed. Meaning, the polarity of the genes’ expression response (repression/activation) is not correlated to whether the signal is disrupted by removing the hormone or the receptor exclusively. This indicates the ability of the receptor to act upon gene regulation, even gene activation, is not completely dependent upon hormone pulse, and vice versa. Otherwise, we would observe only down-regulation of target genes upon hormone or receptor removal. This suggests that the ecdysone signal is more modular, with separable responses among the hormone, ligand bound and unliganded receptor. Below, we categorize the ecdysone and EcR responsive genes as common or shared target genes and exclusive target genes as we further dissect the modules of target genes.

### Common target genes, sensitive to both hormone and receptor loss at pupariation onset

**Fig. 4:**
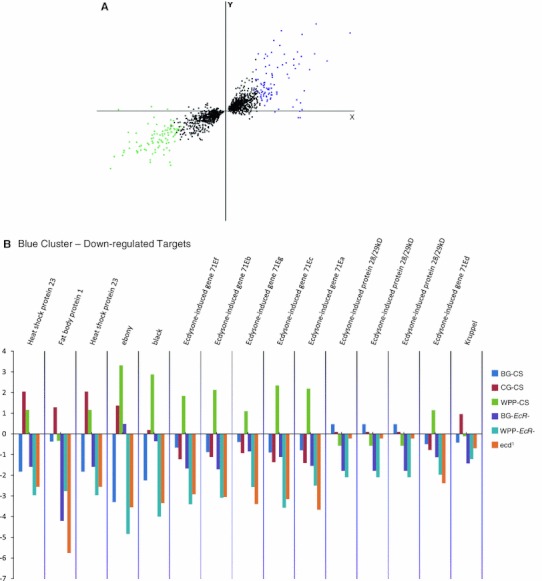

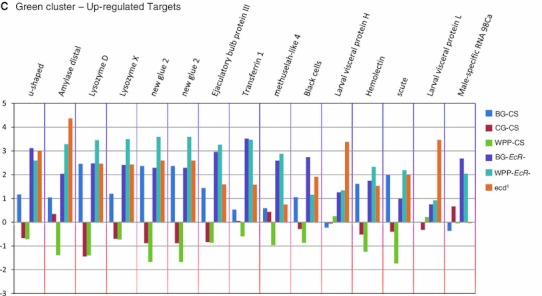
Common, same polarity response genes with the highest differential are well-known ecdysone target genes. **a** Principal component analysis plot of the common, same polarity genes. The colored clusters depict genes which are the most significantly upregulated genes (*green*) or downregulated (*blue*) and have the highest fold changes among the significant genes. **b** Fold changes of the PCA blue cluster genes are shown. These genes represent ecdysone targets that are normally activated at the onset of metamorphosis and include some historically well-known inducible targets, many of which are named as such. The *bar* graphs represent the fold change values for each sample category (*B* and *C*). Fold changes are base on the mutant samples compared to their matched wild type stage while the CS samples where compared to the global mean reference and each are color-coded as indicated. **c** Fold changes of the PCA green cluster genes are shown. These genes represent ecdysone targets that are normally repressed at the onset of metamorphosis and also include some historically well-known repression targets, such as the glue genes and other larval salivary gland proteins

At the WPP stage 592 genes were affected by both EcR and ecdysone removal. Of this common set of differentially expressed target genes, 204 genes are repressed in both mutants and 293 genes are activated in both mutants (Fig. 2a). This indicates that over 80 % of the common target genes have similar transcriptional response to either removal of the receptor or the hormone when compared to wildtype (Fig. 2b). A Principal Components Analysis (PCA) plot (Fig. 4) allows us to determine the magnitude of the transcriptional responses. The most significant genes are color-coded in Fig. 4, with green representing the up-regulated genes and blue representing the down-regulated genes. These genes most likely represent genes that are the traditional ecdysone-EcR direct targets. In Fig. 4b we show the contrasts of expression among all the tested conditions for the most significant common activation target genes (*p* value <0.001). You can clearly distinguish that the normal expression dynamics of these genes in wildtype expression is severely disrupted by removal of the hormone signal, whether by hormone or receptor removal. In this subset, the normal response of activation is replaced with severe repression of gene expression. These genes are in fact some of the most famous representatives of the so-called direct EcR targets, which are historically categorized as early or late genes in the Ashburner model(Ashburner 1972a, 1975; Ashburner et al. 1974; Huet et al. 1995). In fact, a significant portion of these genes were initially identified due to ‘ecdysone puffs’ and so named for their sensitivity to the hormone (e.g. “Ecdysone-induced gene 71Ea, b, c, b, f… etc.) (Supplemental Table 2a). Gene Ontology enrichment analysis of these common targets shows several categories of known ecdysone regulation; including “Late Puff ecdysone regulated protein” (adjusted *p* value 0.01, FDR of 0.8 %) and “pigmentation during development” (raw *P* value 0.00657, FDR of 8 %) (Table 2). Likewise, the common repression target genes are also enriched with classical ecdysone targets including the glue genes (Table 2 and Supplemental Table 2b). Figure 4c shows the relative expression levels for a subset of these genes, in all categories analyzed. Again, we can clearly distinguish that the normal response in wild type is severely impacted by the loss of the hormone signal. In this case, the genes become aberrantly activated.

### Genes regulated by the steroid hormone, ecdysone

For the *ecd1* experiment we compiled a list of ecdysone target genes that were responsive to the removal of the steroid signal across the onset of pupariation (Table 1, 2). Our primary comparison (Fig. 1c, hyb (1) between *p*-*ecd* ^*1*^ and *r*-*ecd* ^*1*^ yielded 1,621 responsive genes. However, based on our experimental design, we anticipated this list included two responsive gene categories. The first and most pertinent category is a response to the removal of the hormone (Henrich et al. 1993); the second would be a heat induced response due to temperature shifting. The heat response was addressed with hyb 2 in our experimental design, CS-22C vs. CS-29C (Supplemental Fig. 2). Forty-three genes were identified as heat response artifacts (Supplemental Fig. (2). However, there were no overlaps with the *ecd1* gene list (Supplemental Fig. 5) and with so few gene hits in this category our data suggests that there was not a significant contribution of heat stress to create a bias in our final set of ecdysone target genes. Additionally, we confirmed the these findings with hyb 3 in our experimental design, *r*-*ecd* ^1^ versus WPP-CS-29C conditions, which directly removed any heat effects to ensure our non-overlapping gene list findings and validate the hormone response target findings. We found there was a high level of variation in this comparison, likely due to genomic background differences. This high level of variation precluded our ability to identify genes that passed the statistical cutoff. However, even upon reducing the statistical threshold (to *p* = 0.05 with 10 % FDR), we found that all significant genes were a subset of the previous *ecd* ^*1*^ comparisons. To address concerns of significant genomic background modifiers between the wildtype and permissive mutant lines, hyb 4, *p*-*ecd* ^*1*^ versus CS-22C, revealed that at 5 % FDR there are 162 significant genes in this comparison (158 up-regulated in the *ecd* ^*1*^ strain and 2 down-regulated) which indicates there is a detectable difference in homeostatic gene expression trends between the two fly strains. This difference in expression levels between the two strains in their non-manipulated wild type state echoes the noise measured in hyb 3, the experimental *r*-*ecd* ^*1*^ versus CS-29C comparison above. However, of these significant background modifier genes, only two overlapped with the previous *ecd*-*29* versus *ecd*-*22* comparisons defined above (Supplemental Fig. 5). Therefore, we conclude there isn’t a significant contribution of genomic background bias within the confirmed ecdysone target gene list. Ultimately, the systematic biases we expected to occur were confounded in the experimental design. Therefore, we define the *ecd1* or ecdysone responsive gene list as the genes found to be significant in the *p*-*ecd* ^*1*^ versus *r*-*ecd* ^*1*^ comparison (Supplemental Table 3).

**Fig. 5:**
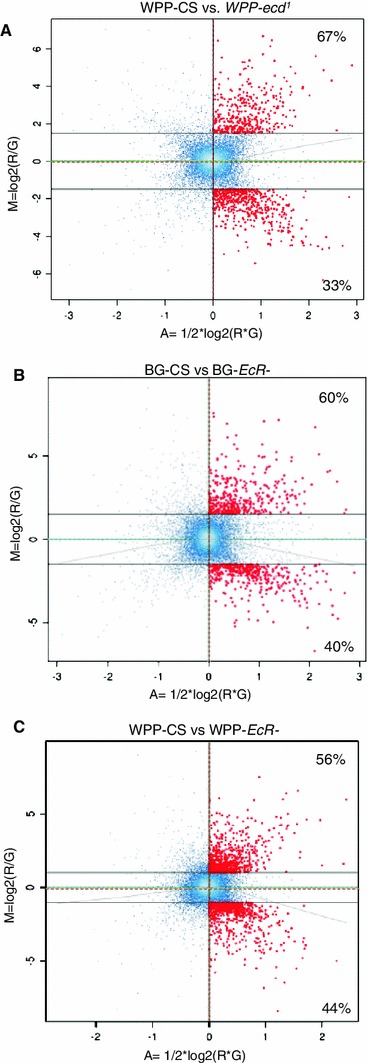
Differentially expressed genes among stage-matched pairs of *EcR*- and *ecd* ^*1*^ mutants. Graphs shown are MA plots used to evaluate the relative gene expression of stage-matched comparisons, displaying the signifcant differentially expressed target genes for each respective comparison. The *red* (R) and *green* (G) lines delineate the global mean of each mutant and reference/control sample, respectively. The *X* and *Y* axes represent the median average global expression values (A) and log2 ratios as regulation values (M) of all genes, respectively. The *grey colored line* corresponds to the loess fit curve. *Red* marked points represent the genes that were found to be statistically significant in the comparison. Points are also color-coded for density of probes, *white* coloring indicates high density, *blue* indicates low density. **a** WPP-CS versus *WPP*-*ecd* ^*1*^ mutant. 1,021 genes were defined to be significantly different in this paired analysis. Of all significant genes, 67 % were up-regulated while 33 % where down-regulated. **b** BG-CS versus BG-*EcR*-*l*. *Red* points indicate genes with significantly different expression levels between these two conditions. This set of targets represents genes which require the unliganded receptor at BG for normal gene expression regulation. 975 of the genes were defined to be significantly different in this paired analysis. **c** WPP-CS versus WPP-*EcR*-. 1,588 of the genes were defined to be statistically different in this paired analysis. These genes represent target genes which require the liganded WPP (*post-hormone pulse*) ecdysone receptor

Due to possible immeasurable issues with developmental de-synchronization between the mutant and wildtype categories, we filtered our target gene list to include only the genes that are normally dynamic during the onset of metamorphosis. Accordingly, the list was reduced to 1,021 by filtering for genes which were also significant in a pairwise comparison to the wild type WPP stage (Fig. 5a). With a 5 % False Discovery Rate (FDR) we find that of the 1,021 genes with significant changes across metamorphosis onset, 33 % of were down-regulated in the mutant condition and 67 % were up-regulated (Fig. 5a), indicating the majority of genes that respond to the hormone signal are repression targets or tightly regulated to control the magnitude of their expression.

**Fig. 6:**
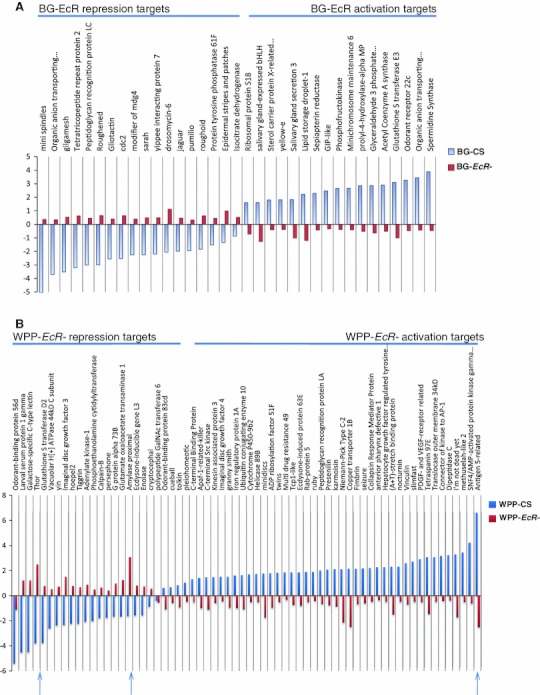

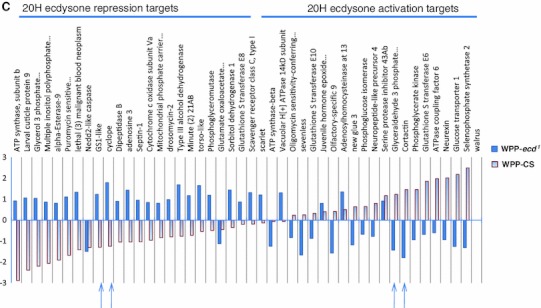
Exclusive metamorphosis target genes, specific to either unliganded receptor, ligand bound receptor or hormone. *Bar* graphs display the fold changes of gene expression for target genes exclusive to each indicated mutant condition. These subsets are also dynamic in wild type expression (metamorphosis onset genes). The *CS bars* represent the normal gene expression change at the relevant stages (relative to mean expression across BG, CG, and WPP stages). **a** The most significant BG-EcR- exclusive target genes. Presence of both activation and repression targets suggest the BG, pre-hormone receptor does not solely play a repressive role, but is also necessary for active transcription. **b** Subset of target genes exclusive to removing the EcR at WPP. Removing the receptor at the WPP stage also results in most genes showing a reversal in normal expression changes. A few also have significant reverse polarity effects at this stage (*arrows*). **c** Subset of target genes exclusive to removing the 20 H ecdysone. It appears that removing the hormone at the WPP stage (ecd) results in most genes losing not only the normal expression change, compared to wild type of either repression or activation, but have significant reversed polarity effects in transcriptional activity at this stage (arrows)

Intriguingly, there are 731 genes regulated by the hormone that are not regulated by the receptor overall. Most of these genes are not normally dynamic at the onset of metamorphosis and these most likely represent some downstream or pleiotropic physiological effects of removing the hormone or the *ecd* ^*1*^ mutant itself. However, upon filtering, the subset of 178 genes within the metamorphosis gene list reflects genes that are responsive to the hormone and not to the receptor at the onset of metamorphosis. This exclusive set of hormone responsive genes includes metabolic and immune response genes Fig. 6c.

### Genes regulated by the nuclear receptor, EcR

From the *EcR*- mutant experiments (Li and Bender 2000), we compiled a list of genes that were responsive to the removal of the ecdysone receptor. By using a common reference design (Fig. 1d), we were able to discern the dynamics of EcR regulation across the onset of metamorphosis (pre and post hormone pulse). Due to the nearly undetectable levels of circulating ecdysone at the BG stage (Warren et al. 2006) when our BG-*EcR*- animals were collected, inherently, the transcriptional profile of the mutant animals reflect changes in gene expression due to removing what is traditionally thought of as an inactive or unliganded (pre-hormone pulse) receptor (i.e. the receptor’s activity prior to the activating effect of ligand binding). Therefore, we interpret the transcriptional response detected in this profile as a reflection of unliganded EcR gene regulation. The WPP-*EcR*- animals were collected at a time point that correlates with 2–4 h past the large pupariation pulse during wild type development. Hence, the transcriptional profile of these animals reveal the change in gene expression due to removing an activated, ligand bound (post-hormone pulse) receptor.

In our initial mixed model analysis we identified, that with a 5 % FDR cutoff, 1,076 genes are differentially regulated in absence of the unliganded BG stage EcR. We also find that with the same statistical cutoff, 1,806 genes have expression changes in response to removing the WPP stage EcR. This 68 % increase in the number of genes relative to the BG mutants, indicates the ligand bound receptor is much more active in transcriptional regulation than the unbound receptor. In total, 2,451 genes are sensitive to removing the EcR, regardless of ligand activity.

To measure the differential gene expression for the explicit mutant timepoints, (BG and WPP) we conducted pair-wise comparisons between the EcR mutants and wild type animals at their comparable stages of development (i.e. BG-*EcR*- compared to BG-CS and WPP-*EcR*- compared to WPP-CS). At the BG stage, 975 genes are regulated by the pre-hormone pulse or unliganded receptor. Of these, 60 % are up-regulated targets, normally repressed in the presence of the receptor at this stage, and 40 % are down-regulated targets, normally activated due to presence of the receptor at this stage (Fig. 5b). At the WPP stage 1,588 genes are regulated by the ligand bound receptor and we observed that 56 % of the WPP-*EcR*- target genes are up-regulated and 44 % are down-regulated (Fig. 5c). Again, this significant increase in the number of responsive genes between BG and WPP indicate the transcriptional regulation activity of the hormone receptor almost doubles upon increase of ecdysone titers and the onset of metamorphosis. This corresponds with numerous published observations of reporter target genes showing inducible transcriptional activity upon presence or increase of the steroid (Kozlova and Thummel 2002; Roth et al. 1999; Warren et al. 2006).

As we compare the differences between the target gene lists of the ligand bound and unliganded receptor, interestingly, we find there are target genes exclusive to each (Fig. 2). Explicitly, there are 108 metamorphosis genes exclusive to the unliganded EcR and 223 genes exclusive to the ligand bound receptor. A closer look at the most significant genes in each category uncovers the biological processes impacted. Figure 6a shows the fold changes, compared to wild type expression change at this time point, for the most significant (*p* = 0.0001) genes for the unliganded receptor. These genes include repression targets that normally function in transcription regulation and morphogenesis and activation targets that include a classical set of genes in this category, salivary gland proteins, which are expressed and retained until pupariation occurs in response to the hormone. In addition, Fig. 6b shows the fold changes for the most significant genes for the ligand bound receptor, compared to wild type, which includes genes that become either repressed or activated post-hormone pulse. These genes include larval proteins and imaginal disc proteins respectively. This correlates with the transition of larval to pupal stages upon onset of metamorphosis and reflects our capacity to detect relevant target genes, even genes named for their ecdysone responsiveness at this stage in development (Fig. 6b).

**Fig. 7:**
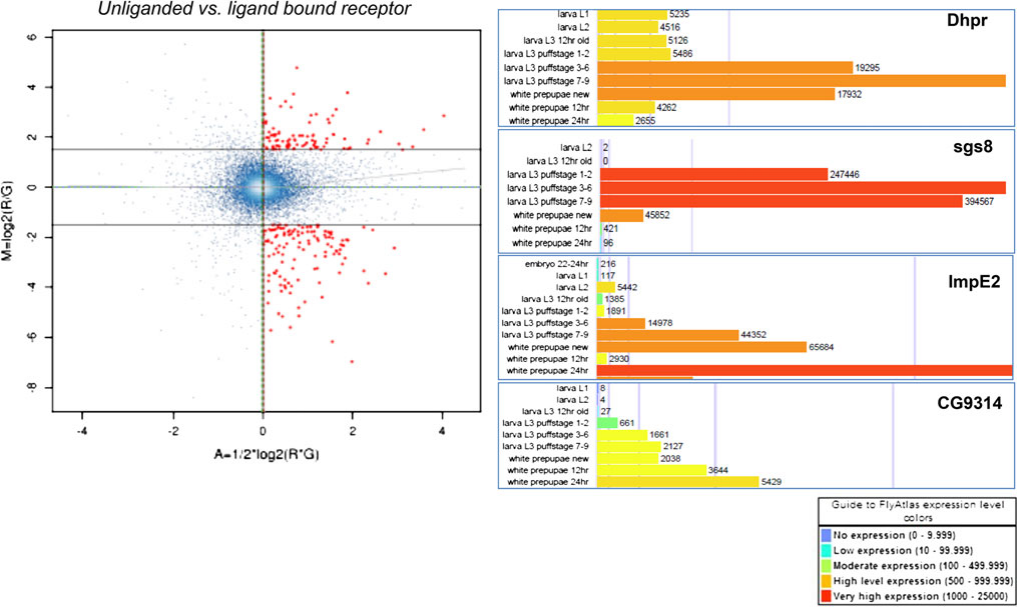
MA plot comparing pre-pulse and post-pulse hormone receptors. 274 genes were defined to be differentially regulated between BG-*EcR*- and WPP-*EcR*-. These genes represent those which are significantly differentially regulated by what we consider the unliganded receptor and the liganded receptor. Most of these genes display opposite polarity in gene expression (Fig. 8). On right, modified images from the FlyAtlas data in FlyBase. The bar graphs represent the indicated genes’ expression levels throughout the lifecycle time course at indicate time points. Each target gene shows an increase at the onset of metamorphosis, which corresponds with our findings implicating ecdysone regulation

**Fig. 8:**
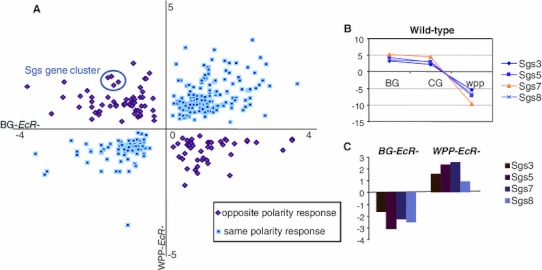
A subset of common EcR-target genes show opposing polarity of regulation. **a** Scatter plot of BG-*EcR*-and WPP-*EcR*- common target genes’ fold changes. The fold change of BG-*EcR*-relative to BG-CS expression is plotted on the *X axis*. The fold change WPP-*EcR*- relative to WPP-CS expression is plotted on the *Y axis*. **b** A *line* graph displaying the normal expression pattern of a set of opposing polarity response targets, the sgs gene cluster, at the onset of metamorphosis in CS. These genes are first upregulated at BG and CG and then are actively repressed across the onset of metamorphosis at WPP. **c** A *bar* graph showing the opposing response of these genes upon the removal of the pre-pulse EcR (BG-*EcR*-) causes a downregulation of the gene cluster at a time point they are normally activated. Conversely, removal of the post-pulse EcR (WPP-*EcR*-) causes upregulation of the gene cluster at a time point they are normally repressed. These data indicate that the ligand binding status of the receptor can impact whether specific target genes are repressed or activated, albeit regulated by the receptor in both bound and unbound states

While there are some distinct targets exclusive to the ligand bound or unliganded receptor, there is also a large overlap between the two *EcR*-categories (Fig. 2), indicating the receptor regulates many of the same genes pre-hormone pulse (unliganded) as post-hormone pulse (ligand bound). An investigation of the common EcR target genes reveals that the polarity of transcriptional response is not always the same between the unliganded and ligand bound receptors for these common target genes (Fig. 7). Specifically, 531 genes are common targets between the pre and post-hormone EcR, 134 are shared up-regulated and 123 are down-regulated. The remaining 274 genes have opposite polarity in gene expression changes. We anticipate such a finding, as an example of genes that are actively repressed by the receptor and then become actively stimulated upon ligand binding, and vice versa. Figure 8b, c show a subset of classical ecdysone targets (SGS genes) that are known to be actively transcribed, under regulation of EcR, and then repressed at the onset of metamorphosis.

A direct paired-test comparison between the ligand bound and unliganded receptors revealed the extent to which genes are differentially regulated between the receptor states (Fig. 7). There are approximately 274 genes in this category. These data indicate that while the receptor actively regulates target genes both pre and post-hormone pulse, the ligand binding of the receptor induces a reversal in gene expression for over half of these. Expression levels of these genes throughout the lifecycle also correlate with the hormone pulse. Figure 8 shows expression patterns for several of these genes with a restricted peak of expression around the time of the ecdysone pupariation pulse. These data are modified images, extracted from FlyBase modENCODE data (Flybase ID: FBrf0212041). Specifically, Dhpr and sgs8 are down-regulated in BG-*EcR*- mutants but up-regulated in WPP-*EcR*-. Their normal lifecycle expression includes a peak of induction prior to the onset of metamorphosis, at which point they are extremely repressed within a matter of hours. Our results indicate that these genes are normally actively repressed by the hormone receptor at the point of ligand binding. Conversely, ImpE2 and CG9314 represent genes that are up-regulated in BG-*EcR*- mutants, but down-regulated in WPP-*EcR*-. Their normal lifecycle expression shows a dramatic increase across the pupariation phase. This indicates that these genes are normally induced to increased expression in response to the ligand, via the receptor.

## Discussion

### Advantage of a conditional mutant system

Many of the time-honored ecdysone target genes were identified in classic studies using salivary gland, polytene chromosome, hormone washes and were the basis of deriving the current model of ecdysone signaling, “The Ashburner Model” (Ashburner 1972a, 1975, Ashburner et al. 1974). Recently, investigators have attempted to uncover whole animal as well as tissue specific ecdysone target genes using various in situ techniques including whole animal or organ cultures and cell lines (Andres and Thummel 1992; Huet et al. 1993; Thummel 2002). While each of these approaches are technically plausible to uncover target genes, they lack the benefit of investigating the hormone signal within a viable living system, in vivo. In addition, the technical constraints of these procedures require the use of hormone concentration levels that are tremendously higher than what is ever present in the animal. Similarly, the use of a knockdown system to remove the hormone system is notorious for incomplete and non-homogeneous removal of the receptor. Lastly, using dissected organs removes the true ‘in vivo’ context and indeed, steroid hormone signaling is a textbook example of systems biology where the whole organism displays an intricate cascade of interactions and feedback from tissue specific mechanisms. Therefore, our approach is considered the ideal mode of discovery for accurately delineating the modular signaling components between hormone and receptor gene regulation. Our approach allows us to study the signal within the natural environment, with all other growth and developmental signals and interactions intact. Therefore, we present this conditional mutant analysis as the optimal approach in elucidating genomic and systemic effects of hormone signaling, particularly for deconstructing hormone signals within specific life cycle timepoints. As the ecdysone signal elicits different responses at different timepoints we submit the responses in this work to be specific to the onset of metamorphosis. A full life cycle analysis is warranted and necessary to determine distinct ecdysone targets across all ecdysone pulses.

### The modular hormone signal–receptor versus hormone

Understanding the dynamic mechanisms of steroid gene regulation, including receptor or hormone dependency and independency, is an important aspect of fully elucidating a hormone signaling network and is especially significant when considering clinical applications or caveats of hormone therapies. By virtue of our experimental design, we are capable of determining each module of hormone regulation, including; exclusive sets of genes that are specifically sensitive to the hormone, the unliganded receptor or the ligand bound receptor. Our common set of ecdysone responsive targets (overlaps between the receptor and hormone target genes) are the intersection of target genes that reflect the traditional linear hormone action, as often represented in the Ashburner model for ecdysone signaling. In this model, gene regulation requires both the receptor and the hormone (a ligand bound receptor). However, our findings illustrate there are exclusive sets of gene targets independently regulated by either the hormone or the ligand bound receptor and most intriguingly, the unliganded receptor. These separable modules of steroid gene regulation depict the multifaceted nature of hormone signaling cascades. We have confirmed scores of target genes that were previously identified through genetic interaction analyses within each of the ‘exclusive’ groups (hormone or receptor) as well as among the common/shared gene targets. Gene functions of the common targets show an increased enrichment of biological pathways already known to be regulated by the hormone. These ‘higher order’ GO pathways were not enriched in any of the exclusive gene sets independently, which further supports the necessity to refer to genes retrieved in both sets (inclusively) when defining overall hormone functions. For instance, in Table 1 we see an enrichment in the common “Ecdysone Regulated Genes” (*p* value, 0.047) that was not present in any other separate mutant target gene lists.

We have found over 900 genes which appear to be regulated by the hormone but not by the receptor. 178 of these are also a subset of our metamorphosis onset genes, indicating these are not just aberrant or pleiotropic *ecd* ^*1*^ gene responses, but normally dynamic genes at pupariation. While appearing to have gene expression function exclusive of the receptor, to be regarded as true gene regulation, the steroid’s signal must be transduced to the genomic level via some transcriptional regulator, such as has recently been postulated (Johnston et al. 2011). It is plausible that it may occur through some other receptor pathway that is not commonly viewed as being hormone regulated, i.e. a G-coupled receptor cascade not previously associated with the steroid (Garbuzov and Tatar 2010; Johnston et al. 2011; Mosallanejad et al. 2010; Soin et al. 2010). Therefore, the receptor independent genes (hormone sensitive but not receptor sensitive) may represent genes that are regulated by an alternate receptor that binds or is otherwise activated by the hormone via other chromatin remodeling or epigenetic mechanisms, such as miRNA effects (Garbuzov and Tatar 2010). In addition, most of these genes show functional enrichment in mitochondrial metabolic functions (Table 4), which may implicate the steroid’s direct impact on the mitochondrial genome. Such regulation would not require transduction to the nuclear genome. This is an intriguing hypothesis and warrants investigation into ecdysteroid interactions with mitochondria and the mitochondrial genome.

The set of hormone independent genes (receptor sensitive but not hormone sensitive) represent targets that are regulated by the receptor without need of the actual steroid. This regulation could be accomplished through tethering to other transcription factors or activity via alternate signaling pathways such as protein modifications (i.e. phosphorylation). The gene functions from this category include cytosol and membrane functions, such as ribosomal subunits (*p* value 0.008) and proteosome function (*p* value 0.0067). In the BG-*EcR*- exclusive category includes enrichment of protein transport (*p* value 0.003), while endocytosis (*p* value 0.01) is enriched in the WPP-*EcR*- exclusive category. These functions may be clues to the non-genomic effects of the hormone signal (Blackmore 1993; Bramley 2003; Groeneweg et al. 2011; Losel et al. 2002; McEwen 1991; Morimoto et al. 2010; Rafiq et al. 2011; Wehling and Losel 2006) and may implicate the receptor in alternate functions and physiological interactions outside of direct gene regulation. However, because we are measuring gene response, these functions must still be modulated on the transcriptional level. In all accounts, these receptor sensitive genes would still be considered to be hormone targets by virtue of their functional association with the hormone receptors. Simply, the regulation exhibited by the hormone receptor function may at times be autonomous, relative to the hormone.

### Implicated biological functions at onset of metamorphosis, proven to be ecdysone regulated

What we often observe upon disruption of the ecdysone hormone signal is a global systemic shutdown of everything. Discerning what systems are regulated by the hormone versus what requires the hormone regulated systems to be functional is somewhat tricky. At a time of development when everything seems to be changing, it is dangerously convenient to simply imply that everything is regulated by the demarcating temporal signal, which in this case is a large pulse of ecdysone. This is why it was important to include in our investigation a parallel study of the wild type gene changes at this time point, not only in reference to gene expression changes in the mutant, but independently showing the gene expression changes in the wild type animal.

To translate the ecdysone responsive gene lists into biologically significant information, we conducted a gene function enrichment analysis. We found that the prevailing general categories are those dealing with catalytic metabolism. Specifically, many of these pathways are implicated in the more general categories of biological processes dealing with salivary gland development or glue synthesis as well as signal transduction, including G-coupled receptor function, and cell death. However, simply using separate mutant gene lists in testing for enrichment of biological pathways did not yield the traditional ecdysone regulated categories, termed in the more complex GO families of biological mechanisms, such as molting and pupal development (in other words not the parental terms in the hierarchy), but rather we find the specific biochemical processes that are required for the proper molecular function of these processes. However, when combined into overlapping gene lists or inclusive gene lists from all mutant categories, we do see enrichment in pupal functions; molting cycle genes as well as cell death (Table 5) which are all previously implicated in ecdysone regulation and validates our method of indexing these ecdysone target genes.

Several of the biological functions and gene networks we identified have been previously suspected or indirectly implicated to be ecdysone regulated but definitive target genes were not previously produced. A couple of these functions include, 17 genes which regulate neuron projection morphogenesis (*p* value 0.0018), 9 genes in axon guidance (*p* value 0.01), and 9 genes in neurotransmitter transport (*p* value 0.008), four genes of which deal with neurotransmitter secretion (SNAP, HRS, usnp and sec15) and two of which deal with neurotransmitter metabolism (specifically dopamine regulation; e and ple). The HRS (Hepatocyte growth factor Regulated tyrosine kinase Substrate**)** gene is a major player in the role of neurotransmitter regulation and it is of particular interest to us as the solitary peak of expression for this specific gene during all of drosophila development is directly at the onset of metamorphosis (modEncode data) implicating a novel function as a specific regulator of the onset of morphogenesis. While it has been well established that Central Nervous System remodeling and Peripheral Nervous System establishment is ecdysone regulated (Brown et al. 2006; Schubiger et al. 1998), specific genes required for these morphological changes were largely undetermined. We now present several potential direct gene targets that result in the movement and architecture of neuron processes never before shown to be specifically ecdysone regulated. Additionally, the enrichment in Peripheral Nervous System development genes, including the gene *spdo* **-** which functions in cell migration and requires proper tubulin functionality (via microtubulin filaments) that previously has been suggested to be ecdysone regulated (Jochova et al. 1997) has now been implicated as a direct target of the ecdysone pathway.

### Functional enrichment in *ecd*^*1*^ and *EcR*-responsive genes

Genes showing exclusive *ecd* ^*1*^ *sensitivity* (not responsive to loss of EcR) include what appears to be EcR independent activities including, oxidative stress responses (*p* value 2.08^E−19^) and NADH hydrogenase activity (*p* value 8.41^E−09^). When considering the polarity of gene expression changes, we see down-regulation of imaginal disc and cell signaling genes, with upregulation of metabolic and cell adhesion genes (Supplemental Table 3). This correlates with the expected molecular response when imaginal disc differentiation is disrupted and the reduced organ tissue integrity which normally occurs at the onset of metamorphosis is no longer observed when the ecdysone signal is removed. An example of this has been previously described as ‘persisting salivary glands’ past the WPP timepoint in EcR isoform mutant analysis (Bender et al. 1997; Davis et al. 2005).

Alternatively, functional enrichment of genes showing exclusive EcR sensitivity, which appear to be ecdysone independent, include such activities as, ribosomal and protein localization functions. Specifically, the pre-hormone pulse receptor has distinct enrichment for axon guidance (*p* value 0.019) and dendrite development (*p* value 0.06), while the post-hormone pulse receptor regulates such activities as, membrane mediated transport (*p* value 3.37^E−05^) and egg chamber formation (*p* value 0.01).

For our inclusive EcR mutant categories, we see an enrichment of known ecdysone functions, such as programmed cell death (*p* value 0.0016) and autophagic cell death (*p* value 0.02) (Table 3). Also, two of the strongest enrichments occur in gene networks related to proteosome function (*p* value 3.37 ^E−04^) and protein localization (*p* value 3.18^E−04^). This indicates the regulatory capacity of the receptor extends beyond transcriptional modulation, into the integrity and turnover of gene products. This might be expected when we consider the nature of succinct and abrupt physiological changes which occur within minutes of a hormone signal. On top of shutting down gene expression, the residual gene products must be ablated in order to redirect the morphological course of a specific tissue. Here, we present the first signs of how this may be regulated by a steroid hormone, via specific proteosome and protein modification regulation.

When considering the polarity of the transcriptional response in the inclusive EcR categories, we see a significant down regulation of gene functions dealing with organogenesis (Tables 1, 2, 3, and 4) which correlates with the halt of metamorphoses upon removing the hormone signal. Specifically, several genes that are required for imaginal disc development and eversion are down-regulated, including impE2 (Table 6) and imaginal discs growth factor 3 (Fig. 6b).

**Table 6:** Mutant *EcR*- opposite polarity response genes

Mutant *EcR*-opposite response genes		
Flybase ID	Gene symbol	Biological function
FBgn0000547	ed	Epidermal growth factor receptor signaling pathway
		Cell adhesion
		Negative regulation of epidermal growth factor signaling
		Negative regulation of neurogenesis
		Sensory organ development
FBgn0001254	ImpE2	Imaginal disc eversion
FBgn0004646	Ogre	Phototransduction
		Visual behavior
		Nervous system development
		Signal transduction
FBgn0010229	Hr39	Regulation of transcription, DNA-dependent
		Female meiosis chromosome segregation
		Regulation of transcription from RNA pol II promoter
		Signal transduction
FBgn001159	Grp	Protein amino acid phosphorylation
		DNA damage checkpoint
		Regulation of progression through syncytial blastoderm mitotic cycle
		Cell cycle arrest
		Cellularization (sensu Metazoa)
		Embryonic development (sensu Insecta)
		Imaginal disc development
		Female meiosis chromosome segregation
FBgn0016930	smi35A	Nervous system development
		Response to chemical stimulus
		Protein amino acid phosphorylation
		Cell proliferation
		Ectoderm development
		Induction of apoptosis
FBgn0032061	CG9314	Defense response
		Oxygen and reactive oxygen species metabolism
		Electron transport
		Response to oxidative stress
FBgn0035089	Phk-3	Metamorphosis (sensu Insecta)
		Oogenesis (sensu Insecta)
		Signal transduction
		Gastrulation
		Determination of anterior/posterior axis, embryo
		Ovarian follicle cell development (sensu Insecta)
		Ras protein signal transduction
		Spermatogenesis

This subset of genes are up-regulated in BG-*EcR*- mutants but down-regulated in WPP-*EcR*

**Table 7:** Mutant *EcR*- opposite polarity response genes

Mutant EcR-opposite response genes, cont’d		
Flybase ID	Gene symbol	Biological function
FBgn0002563	Lsp1beta	Transport
FBgn0003357	Jon99Ciii	Proteolysis, digestion
FBgn0003373	Sgs3	Puparial adhesion
FBgn0003375	Sgs5	Puparial adhesion
FBgn0003377	Sgs7	Puparial adhesion
FBgn0003378	Sgs8	Puparial adhesion
FBgn0034138	RpS15	Protein biosynthesis
FBgn0034225	CG4827	Nucleic acid metabolism
		Nucleotide catabolism
FBgn0034564	CG9344	Nuclear mRNA splicing, via spliceosome
FBgn0035154	CG3344	Proteolysis
FBgn0035165	CG13887	B cell mediated immunity
		Apoptosis
		Intracellular protein transport
FBgn0035781	CG8560	Proteolysis
FBgn0035964	Dhpr	Amino acid catabolism
		Coenzyme metabolism
		Pteridine and derivative metabolism
FBgn0036290	CG10638	Aldehyde metabolism
FBgn0036335	mRpL20	Protein biosynthesis
FBgn0036553	CG17027	Dephosphorylation
		Intracellular signaling cascade
		Carbohydrate metabolism
FBgn0036846	MESR6	Biological process unknown
FBgn0037314	CG12000	Cellular physiological process
		Ubiquitin-dependent
		Protein catabolism
FBgn0037913	CG6783	Transport
FBgn0038083	CG5999	Polysaccharide metabolism
		Response to toxin
		Steroid metabolism
FBgn0039241	CG11089	Purine base metabolism
		Purine nucleotide biosynthesis
FBgn0039581	Moca-cyp	Protein folding
		Protein targeting
FBgn0039835	mRpL32	Protein biosynthesis
FBgn0040954	CG13779	Proteolysis
FBgn0043012	AP-2sigma	Neurotransmitter secretion
		Intracellular protein transport
		Receptor mediated endocytosis
		Synaptic vesicle coating

This subset of genes are down-regulated in BG-*EcR*- mutants but up-regulated in WPP-*EcR*-

EcR target genes showing opposing transcriptional responses between the BG stage and the WPPstage outline an intriguing set of target genes (Fig. 8). These genes are regulated by both the liganded and unliganded receptor, but the transcriptional response is distinct in either case. In Table 6, we see that some of these biological functions, such as imaginal disc eversion, nervous system development and sensory organ development have been previously implicated as ecdysone regulated via EcR. We now know that the regulation mediated by the receptor is both repression and activation, depending upon the ligand state of the hormone. Correlations with EcR binding with and without hormone should further confirm these mechanisms (M.Davis et. al in prep).

### De-regulation of genes by removing ecdysone signaling vs. loss of direct activation or repression

In this paper we have revealed hundreds of de-regulated ecdysone gene targets, defined as those genes that are normally quiescent or static in expression during the onset of metamorphosis (not in our metamorphosis onset gene list); however, upon disruption of the ecdysone signal result in a significant change in expression. We find that the majority of these genes are induced upon removal of the ecdysone signal, as opposed to loss of transcriptional activity which might be expected in the traditional linear hormone signal model. This implies a strong level of marginalizing function in the hormone signal. This function has not been reported in such circumstances as anything but aberrant induction until now. We believe that an integral part of maintaining the potential of a hormone’s signaling is to ensure that only target genes respond. What we may be observing is active dampening of transcriptional response by the hormone receptor, for a novel category of target genes. These target genes are bound by the receptor and prevented from activating, most likely to retain the integrity of temporal and spatial specificity. Potentially, there is a level of gene regulation where transcription levels are stabilized or managed to a strict degree by the hormone signal.

Alternatively, this level of reduction could be achieved through active regulation of miRNA genes. For miRNA genes which are regulated by the hormone signal, loss of the hormone would remove the miRNA expression and therefore removes their repressive action on the responsive genes we have detected. Accordingly, the mRNA targets of the miRNA’s would be more stable and accumulate, whereas normally they would be degraded. We have bioinformatically investigated several miRNA genes which are putative targets of EcR (have EcRE’s within their enhancer/promoter region) and found that the majority of these miRNA genes have EcR regulated genes as their putative targets (data not shown). Similarly, this miRNA hypothesis may also explain why there seem to be a larger number of repression targets than activation targets in a pathway that is largely studied and attributed as a prime mechanism for gene activation exploitation.

### Inclusive target gene functions vs. exclusive hormone or receptor target gene functions: ge ontology enrichment

Upon further investigation of the gene networks we discovered that there is a significant difference in the power of biological function analysis depending on whether we used an ‘overlap’, exclusive or inclusive/cumulative gene lists. The filtered ‘overlapping’ set of differential genes which follows the traditional linear understanding of a hormone response, defined by a filtered correlation of response with either the *ecd* ^*1*^ response and/or the complementary *EcR*- category (Fig. 1), yields qualitatively different functional categories in an GO enrichment analysis than when we used the ‘inclusive’ set of differential gene targets (including all genes with significant expression changes in either mutant category). Ultimately we find that the associations of functional networks related to known ecdysone regulation and/or ecdysone mutant phenotypes are detected more robustly using the ‘inclusive’ gene list for Gene Ontology enrichment calculations (Tables 1, 2, 3, 4 and 5). The exclusive gene lists were useful when trying to parse potential components of these functional networks that may be receptor or hormone independent.

The overlap gene list, for genes significantly changing in all categories, (Table 5) includes some enrichment of cell death and puparial genes, but not all statistically significant. However, the inclusive gene list, including target genes from the ecdysonless and both *EcR*- categories, includes higher enrichment in functions such as molting cycle (*p* value 1.83^E−06^), puparial adhesion (*p* value 4.88^E−07^) and autophagic cell death (*p* value 149^E−05^). We also see emerging GO category functions that were not detected in the separate mutant or overlap gene lists, such as gland morphogenesis (*p* value 7.66^E−05^), glycoloysis (p value 0.001), muscle cell differentiation (p value 0.04) and wound healing (p value 0.004). Conversely, the exclusive gene lists suggest a separation of distinct regulation between the hormone and the receptor.

### Correlations with other whole genome ecdysone and EcR studies

Several studies have been published which directly address ecdysone responsive genes in an *in vitro* context. We conducted a comparative analysis of the most comparable dataset to determine if there is significant overlap of target gene findings. We found that a similar study of target gene analysis has a less than 5 % overlap with our findings in the ecdysone sensitive category. Table 8 shows a summary of the target gene categories from our study. Specifically of the 743 genes published in a previous ecdysone responsive data set (Beckstead et al. 2005), only 44 overlap with our *ecd* ^*1*^ gene set. The experimental design would have been considered the reciprocal experiment of our work (removal vs. addition of hormone); however, the lack of correlation with gene targets creates an air of uncertainty especially when considering their experimental conditions of utilizing an extreme amount of hormone for treatment in a cultured organ environment. We believe that our dataset is more complete and biologically sound in that it was completed using live animals as opposed to laboratory cell lines and artificial organ cultures.

**Table 8:** Summary of responsive genes and measured variables

	Hyb category	Dynamic genes	Up-regulated^a^	Down-regulated^a^	Exclusive targets	Meta-morphosis genes	Exclusive metamorphosis genes	Measured variables
*Ecdysoneless* ^*1*^	1	1621	1201	420	731	1021	178	Ecdysone+heat
	2	43	3	40	n.d	n.d.	n.d.	Heat
	3	133	127	6	n.d	n.d.	n.d.	Gen. background
	4	91	65	26	n.d	n.d.	n.d.	Gen. background+heat
*BG*-*EcR*-	5	1076	459	323	417	367	108	Unliganded receptor
*WPP*-*EcR*-	6	1807	1010	797	726	651	223	Ligand bound receptor
CS-cnbw	Wild type	1255	293	204	263	1255	263	Metamorphosis genes

a In addition to a 5.0 Q-value cut off, only genes with a 1.5 fold change were included in the up or down-regulated gene list

### EcR binding, confirmation of direct targets of ecdysone signaling

**Fig. 9:**
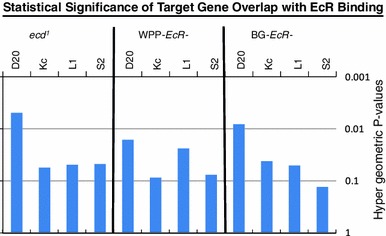
Ecdysone response genes overlap with EcR binding sites. Response genes for the three mutant categories were compared to the binding sites of EcR in four cell lines. Each category has a significant number of genes which overlap with EcR binding targets. Binding target genes were defined as having an EcR binding site within 1 Kb of transcription start site. The EcR binding assays were conducted in four different cell lines including two embryonic lines (Kc and S2) as well as two imaginal disc cell lines (D20 and L1) (Davis and White, unpublished). The hypergeometric pvalue for the overlaps of these respective gene lists is shown

Of our inclusive gene set, there were nearly 230 genes that are confirmed EcR binding targets; defined as having a true EcR binding site within 4 kb of their transcription startsite detected in the Kc cell line using a DamID procedure (Davis et. al, in prep) (Fig. 9). There was no bias in either particular mutant category with confirmed binding from the binding data used. We do see some arbitrary enrichment in bound targets from the WPP-*EcR*- category associated with genes that related to signal transduction and transport, while BG-*EcR*- bound targets are associated with proteasome. There were several known targets confirmed, including Sgs-4, broad, Eip 63E, Eig E1 as well as some suspected targets validated, including Slobo, shaggy, Rab-proteins, nocturin and others. However, there is most likely some other qualitative differences between the regulation potential of pre-hormone target genes and those which only show a response post-hormone pulse. Further investigations which compare the regulatory elements and cofactor interactions of pre-hormone and post-hormone EcR targets would be necessary to address these possibilities and are forthcoming in a genome-wide analysis of dynamic EcR binding sites *(M.Davis and K.White, in prep).*

In conclusion, when considering the variegated responses of the steroid hormone signal, we have presented an indexed catalog of target genes and functions that either require both the hormone and its receptor, or require one, but not the other. These data illustrate independent mechanisms and gene targets which provide evidence of separable hormone receptor functions and lays groundwork for determining these separate functions throughout the lifecycle. It also serves as a model for similar studies in other steroid pathways in higher organisms.

## Electronic supplementary material

Below is the link to the electronic supplementary material.

Supplementary material 1 (PPTX 609 kb)

Supplementary material 2 (XLSX 3469 kb)

## References

[CR1] Africander D, Verhoog N, Hapgood JP (2011). Molecular mechanisms of steroid receptor-mediated actions by synthetic progestins used in HRT and contraception. Steroids.

[CR2] Andres AJ, Thummel CS (1992). Hormones, puffs and flies: the molecular control of metamorphosis by ecdysone. Trends Genet.

[CR3] Apple RT, Fristrom JW (1991). 20-Hydroxyecdysone is required for, and negatively regulates, transcription of Drosophila pupal cuticle protein genes. Dev Biol.

[CR4] Ashburner M (1967). Patterns of puffing activity in the salivary gland chromosomes of drosophila I. Autosomal puffing patterns in a laboratory stock of *Drosophila melanogaster*. Chromosoma.

[CR5] Ashburner M (1969). Patterns of puffing activity in the salivary gland chromosomes of drosophila II. The X-chromosome puffing patterns of *D. melanogaster* and *D. simulans*. Chromosoma.

[CR6] Ashburner M (1970). Patterns of puffing activity in the salivary gland chromosomes of drosophila V. Responses to environmental treatments. Chromosoma.

[CR7] Ashburner M (1972). Puffing patterns in *Drosophila melanogaster* and related species. Results Probl Cell Differ.

[CR8] Ashburner M (1972). Patterns of puffing activity in the salivary gland chromosomes of drosophila VI. Induction by ecdysone in salivary glands of *D. melanogaster* cultured in vitro. Chromosoma.

[CR9] Ashburner M (1974). Sequential gene activation by ecdysone in polytene chromosomes of *Drosophila melanogaster* II. The effects of inhibitors of protein synthesis. Dev Biol.

[CR10] Ashburner M (1975). The genetic and hormonal control of puffing in the salivary gland chromosomes of drosophila. Sov J Dev Biol.

[CR11] Ashburner M, Chihara C, Meltzer P, Richards G (1974). Temporal control of puffing activity in polytene chromosomes. Cold Spring Harb Symp Quant Biol.

[CR12] Beckstead RB, Lam G, Thummel CS (2005). The genomic response to 20-hydroxyecdysone at the onset of *Drosophila metamorphosis*. Genome Biol.

[CR13] Bender M, Imam FB, Talbot WS, Ganetzky B, Hogness DS (1997). Drosophila ecdysone receptor mutations reveal functional differences among receptor isoforms. Cell.

[CR14] Berreur P, Porcheron P, Moriniere M, Berreur-Bonnenfant J, Belinski-Deutsch S, Busson D, Lamour-Audit C (1984). Ecdysteroids during the third larval instar in 1(3)ecd-1ts, a temperature-sensitive mutant of *Drosophila melanogaster*. Gen Comp Endocrinol.

[CR15] Blackmore PF (1993). Rapid non-genomic actions of progesterone stimulate Ca2^+^ influx and the acrosome reaction in human sperm. Cell Signal.

[CR16] Bramley T (2003). Non-genomic progesterone receptors in the mammalian ovary: some unresolved issues. Reproduction.

[CR17] Brown HL, Cherbas L, Cherbas P, Truman JW (2006). Use of time-lapse imaging and dominant negative receptors to dissect the steroid receptor control of neuronal remodeling in drosophila. Development.

[CR18] Bryant Z, Subrahmanyan L, Tworoger M, LaTray L, Liu CR, Li MJ, van den Engh G, Ruohola-Baker H (1999). Characterization of differentially expressed genes in purified drosophila follicle cells: toward a general strategy for cell type-specific developmental analysis. Proc Natl Acad Sci USA.

[CR19] Buijs C, de Vries EG, Mourits MJ, Willemse PH (2008). The influence of endocrine treatments for breast cancer on health-related quality of life. Cancer Treat Rev.

[CR20] Buszczak M, Freeman MR, Carlson JR, Bender M, Cooley L, Segraves WA (1999). Ecdysone response genes govern egg chamber development during mid-oogenesis in drosophila. Development.

[CR21] Carney GE, Bender M (2000). The Drosophila ecdysone receptor (EcR) gene is required maternally for normal oogenesis. Genetics.

[CR22] Cherbas L, Hu X, Zhimulev I, Belyaeva E, Cherbas P (2003). EcR isoforms in drosophila: testing tissue-specific requirements by targeted blockade and rescue. Development.

[CR23] da Huang W, Sherman BT, Stephens R, Baseler MW, Lane HC, Lempicki RA (2008). DAVID gene ID conversion tool. Bioinformation.

[CR24] da Huang W, Sherman BT, Lempicki RA (2009). Systematic and integrative analysis of large gene lists using DAVID bioinformatics resources. Nat Protoc.

[CR25] Dahlquist KD, Salomonis N, Vranizan K, Lawlor SC, Conklin BR (2002). GenMAPP, a new tool for viewing and analyzing microarray data on biological pathways. Nat Genet.

[CR26] D’Avino PP, Thummel CS (2000). The ecdysone regulatory pathway controls wing morphogenesis and integrin expression during *Drosophila metamorphosis*. Dev Biol.

[CR27] Davis MB, Carney GE, Robertson AE, Bender M (2005). Phenotypic analysis of EcR-A mutants suggests that EcR isoforms have unique functions during drosophila development. Dev Biol.

[CR28] Dennis G, Sherman BT, Hosack DA, Yang J, Gao W, Lane HC, Lempicki RA (2003). DAVID: database for annotation, visualization, and integrated discovery. Genome Biol.

[CR29] Doniger SW, Salomonis N, Dahlquist KD, Vranizan K, Lawlor SC, Conklin BR (2003). MAPPFinder: using gene ontology and GenMAPP to create a global gene-expression profile from microarray data. Genome Biol.

[CR30] Doughty JC (2011). When to start an aromatase inhibitor: now or later?. J Surg Oncol.

[CR31] Eberle AN, Mild G, Froidevaux S (2004). Receptor-mediated tumor targeting with radiopeptides Part 1. General concepts and methods: applications to somatostatin receptor-expressing tumors. J Recept Signal Transduct Res.

[CR32] Eigenbrot C, Ultsch M, Dubnovitsky A, Abrahmsen L, Hard T (2010). Structural basis for high-affinity HER2 receptor binding by an engineered protein. Proc Natl Acad Sci USA.

[CR33] Eisen MB, Spellman PT, Brown PO, Botstein D (1998). Cluster analysis and display of genome-wide expression patterns. Proc Natl Acad Sci USA.

[CR34] Fassnacht M, Libe R, Kroiss M, Allolio B (2011). Adrenocortical carcinoma: a clinician’s update. Nat Rev Endocrinol.

[CR35] Gagou ME, Kapsetaki M, Turberg A, Kafetzopoulos D (2002). Stage-specific expression of the chitin synthase DmeChSA and DmeChSB genes during the onset of *Drosophila metamorphosis*. Insect Biochem Mol Biol.

[CR36] Gangadharan C, Thoh M, Manna SK (2010). Late phase activation of nuclear transcription factor kappaB by doxorubicin is mediated by interleukin-8 and induction of apoptosis via FasL. Breast Cancer Res Treat.

[CR37] Ganter GK (2011). Drosophila male courtship behavior is modulated by ecdysteroids. J Insect Physiol.

[CR38] Ganter GK (2012). Drosophila female precopulatory behavior is modulated by ecdysteroids. J Insect Physiol.

[CR39] Garbuzov A, Tatar M (2010). Hormonal regulation of drosophila microRNA let-7 and miR-125 that target innate immunity. Fly (Austin).

[CR40] Garen A, Kauvar L, Lepesant JA (1977). Roles of ecdysone in drosophila development. Proc Natl Acad Sci USA.

[CR41] Gauhar Z, Sun LV, Hua S, Mason CE, Fuchs F, Li TR, Boutros M, White KP (2009). Genomic mapping of binding regions for the ecdysone receptor protein complex. Genome Res.

[CR42] Gaziova I, Bonnette PC, Henrich VC, Jindra M (2004). Cell-autonomous roles of the ecdysoneless gene in drosophila development and oogenesis. Development.

[CR43] Giraudo M, Califano J, Hilliou F, Tran T, Taquet N, Feyereisen R, Le Goff G (2011). Effects of hormone agonists on Sf9 cells, proliferation and cell cycle arrest. PLoS One.

[CR44] Gonsalves SE, Neal SJ, Kehoe AS, Westwood JT (2011). Genome-wide examination of the transcriptional response to ecdysteroids 20-hydroxyecdysone and ponasterone A in *Drosophila melanogaster*. BMC Genomics.

[CR45] Groeneweg FL, Karst H, de Kloet ER, Joels M (2011). Rapid non-genomic effects of corticosteroids and their role in the central stress response. J Endocrinol.

[CR46] Hayes E, Nicholson RI, Hiscox S (2011). Acquired endocrine resistance in breast cancer: implications for tumour metastasis. Front Biosci.

[CR47] Henrich VC, Livingston L, Gilbert LI (1993). Developmental requirements for the ecdysoneless (ecd) locus in *Drosophila melanogaster*. Dev Genet.

[CR48] Henrich VC, Rybczynski R, Gilbert LI (1999). Peptide hormones, steroid hormones, and puffs: mechanisms and models in insect development. Vitam Horm.

[CR49] Hospers GA, Helmond FA, de Vries EG, Dierckx RA, de Vries EF (2008). PET imaging of steroid receptor expression in breast and prostate cancer. Curr Pharm Des.

[CR50] Huet F, Ruiz C, Richards G (1993). Puffs and PCR: the in vivo dynamics of early gene expression during ecdysone responses in drosophila. Development.

[CR51] Huet F, Ruiz C, Richards G (1995). Sequential gene activation by ecdysone in *Drosophila melanogaster*: the hierarchical equivalence of early and early late genes. Development.

[CR52] Jiang C, Lamblin AF, Steller H, Thummel CS (2000). A steroid-triggered transcriptional hierarchy controls salivary gland cell death during *Drosophila metamorphosis*. Mol Cell.

[CR53] Jochova J, Zakeri Z, Lockshin RA (1997). Rearrangement of the tubulin and actin cytoskeleton during programmed cell death in drosophila salivary glands. Cell Death Differ.

[CR54] Johnston DM, Sedkov Y, Petruk S, Riley KM, Fujioka M, Jaynes JB, Mazo A (2011). Ecdysone- and NO-mediated gene regulation by competing EcR/Usp and E75A nuclear receptors during drosophila development. Mol Cell.

[CR55] Kim HS, Freedland SJ (2010). Androgen deprivation therapy in prostate cancer: anticipated side-effects and their management. Curr Opin Support Palliat Care.

[CR56] Kozlova T, Thummel CS (2000). Steroid regulation of postembryonic development and reproduction in drosophila. Trends Endocrinol Metab.

[CR57] Kozlova T, Thummel CS (2002). Spatial patterns of ecdysteroid receptor activation during the onset of *Drosophila metamorphosis*. Development.

[CR58] Lam G, Hall BL, Bender M, Thummel CS (1999). DHR3 is required for the prepupal-pupal transition and differentiation of adult structures during *Drosophila metamorphosis*. Dev Biol.

[CR59] Li T, Bender M (2000). A conditional rescue system reveals essential functions for the ecdysone receptor (EcR) gene during molting and metamorphosis in drosophila. Development.

[CR60] Li TR, White KP (2003). Tissue-specific gene expression and ecdysone regulated genomic networks in drosophila. Dev Cell.

[CR61] Li H, Harrison D, Jones G, Jones D, Cooper RL (2001). Alterations in development, behavior, and physiology in drosophila larva that have reduced ecdysone production. J Neurophysiol.

[CR62] Losel R, Feuring M, Wehling M (2002). Non-genomic aldosterone action: from the cell membrane to human physiology. J Steroid Biochem Mol Biol.

[CR63] McEwen BS (1991). Non-genomic and genomic effects of steroids on neural activity. Trends Pharmacol Sci.

[CR64] Morimoto S, Morales A, Zambrano E, Fernandez-Mejia C (2010). Sex steroids effects on the endocrine pancreas. J Steroid Biochem Mol Biol.

[CR65] Mosallanejad H, Badisco L, Swevers L, Soin T, Knapen D, Vanden Broeck J, Smagghe G (2010). Ecdysone signaling and transcript signature in drosophila cells resistant against methoxyfenozide. J Insect Physiol.

[CR66] Napieralski R, Brunner N, Mengele K, Schmitt M (2010). Emerging biomarkers in breast cancer care. Biomark Med.

[CR67] Rafiq K, Hitomi H, Nakano D, Nishiyama A (2011). Pathophysiological roles of aldosterone and mineralocorticoid receptor in the kidney. J Pharmacol Sci.

[CR68] Rainer J, Sanchez-Cabo F, Stocker G, Sturn A, Trajanoski Z (2006). CARMAweb: comprehensive R- and bio-conductor-based web service for microarray data analysis. Nucleic Acids Res.

[CR69] Riddiford LM, Cherbas P, Truman JW (2000). Ecdysone receptors and their biological actions. Vitam Horm.

[CR70] Roth GE, Wattler S, Bornschein H, Lehmann M, Korge G (1999). Structure and regulation of the salivary gland secretion protein gene Sgs-1 of *Drosophila melanogaster*. Genetics.

[CR71] Schubiger M, Wade AA, Carney GE, Truman JW, Bender M (1998). Drosophila EcR-B ecdysone receptor isoforms are required for larval molting and for neuron remodeling during metamorphosis. Development.

[CR72] Smyth GK, Gentleman R, Dudoit S, Irizarry R, Huber W (2005). Limma: linear models for microarray data. Bioinformatics and computational biology solutions using R and bio-conductor.

[CR73] Smyth GK, Speed T (2003). Normalization of cDNA microarray data. Methods.

[CR74] Soin T, Swevers L, Kotzia G, Iatrou K, Janssen CR, Rouge P, Harada T, Nakagawa Y, Smagghe G (2010). Comparison of the activity of non-steroidal ecdysone agonists between dipteran and lepidopteran insects, using cell-based EcR reporter assays. Pest Manag Sci.

[CR75] Terashima J, Bownes M (2005). A microarray analysis of genes involved in relating egg production to nutritional intake in *Drosophila melanogaster*. Cell Death Differ.

[CR76] Thummel CS (2002). Ecdysone-regulated puff genes 2000. Insect Biochem Mol Biol.

[CR77] Tian L, Guo E, Diao Y, Zhou S, Peng Q, Cao Y, Ling E, Li S (2010). Genome-wide regulation of innate immunity by juvenile hormone and 20-hydroxyecdysone in the Bombyx fat body. BMC Genomics.

[CR78] Toft DJ, Cryns VL (2011). Minireview: basal-like breast cancer: from molecular profiles to targeted therapies. Mol Endocrinol.

[CR79] van den Berg NS, Buckle T, Kuil J, Wesseling J, van Leeuwen FW (2011). Immunohistochemical detection of the CXCR4 expression in tumor tissue using the fluorescent peptide antagonist Ac-TZ14011-FITC. Transl Oncol.

[CR80] Vaquerizas JM, Dopazo J, Diaz-Uriarte R (2004). DNMAD: web-based diagnosis and normalization for microarray data. Bioinformatics.

[CR81] Warren JT, Bachmann JS, Dai JD, Gilbert LI (1996). Differential incorporation of cholesterol and cholesterol derivatives into ecdysteroids by the larval ring glands and adult ovaries of *Drosophila melanogaster*: a putative explanation for the l(3)ecd1 mutation. Insect Biochem Mol Biol.

[CR82] Warren JT, Yerushalmi Y, Shimell MJ, O’Connor MB, Restifo LL, Gilbert LI (2006). Discrete pulses of molting hormone, 20-hydroxyecdysone, during late larval development of Drosophila melanogaster: correlations with changes in gene activity. Dev Dyn.

[CR83] Wehling M, Losel R (2006). Non-genomic steroid hormone effects: membrane or intracellular receptors?. J Steroid Biochem Mol Biol.

[CR84] Wettenhall JM, Smyth GK (2004). LimmaGUI: a graphical user interface for linear modeling of microarray data. Bioinformatics.

[CR85] White KP, Rifkin SA, Hurban P, Hogness DS (1999). Microarray analysis of drosophila development during metamorphosis. Science.

