# Supplementary material for: Genomic analysis of the ecdysone steroid signal at metamorphosis onset using ecdysoneless and EcRnullDrosophila melanogaster mutants
Source: Genes Genomics. 2013 Feb 5;35(1):21–46. doi: 10.1007/s13258-013-0061-0 (PMC3585846; doi:10.1007/s13258-013-0061-0)
Supplement: Supplementary file 1 — Supplementary material 1 (PPTX 609 kb) [file 13258_2013_61_MOESM1_ESM.pptx]

## Slide 1
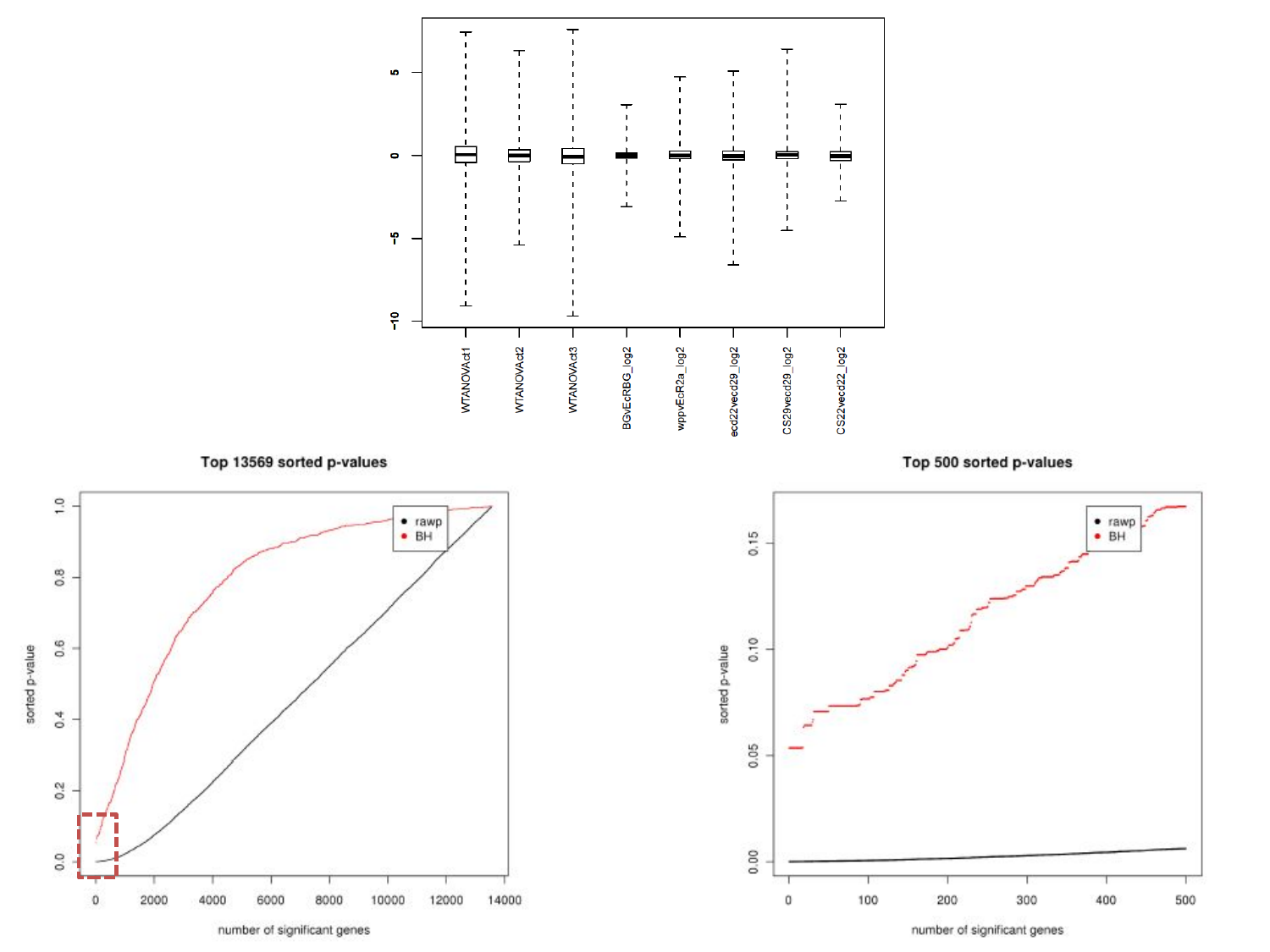

## Slide 2
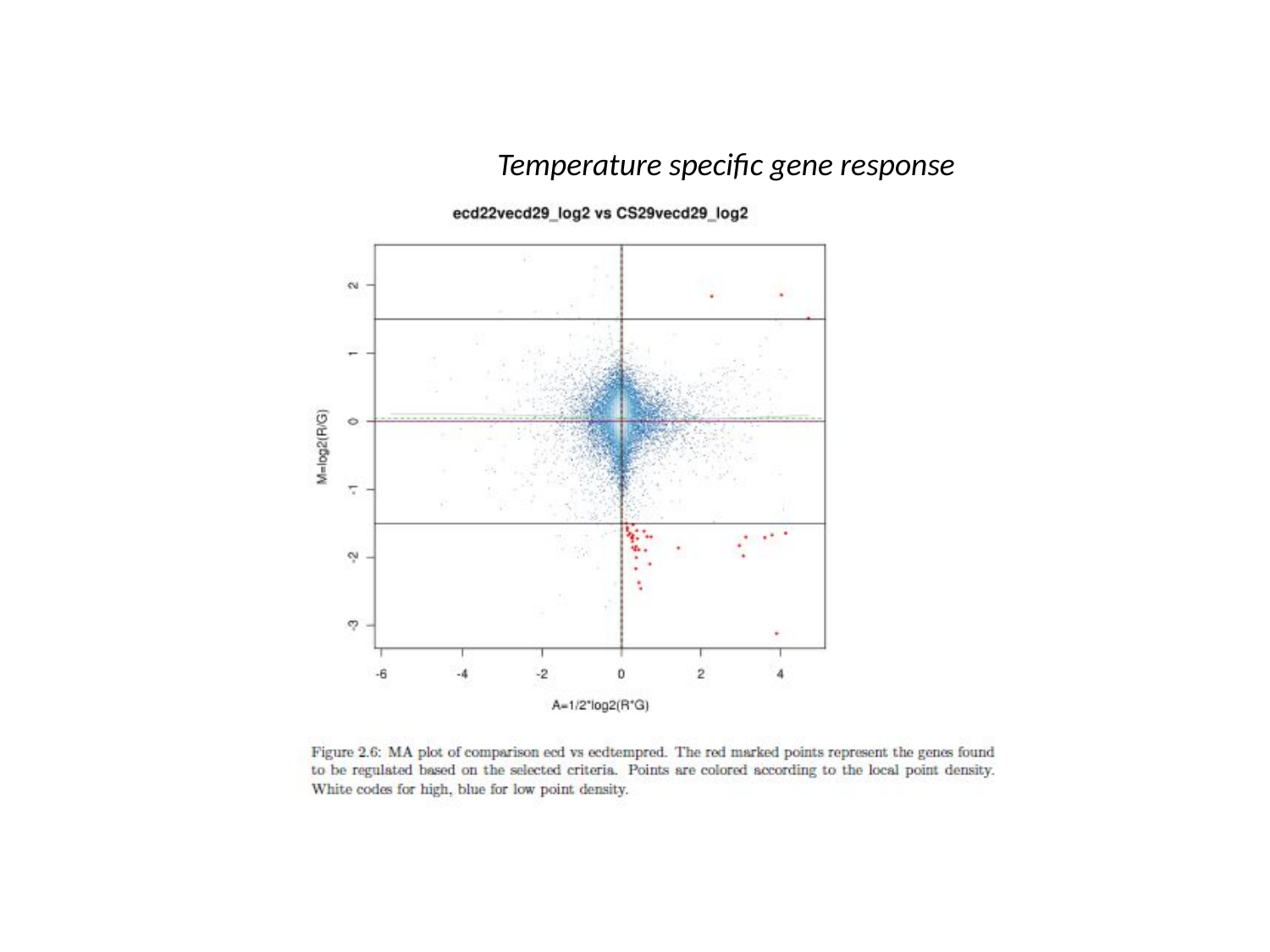

Temperature specific gene response

## Slide 3
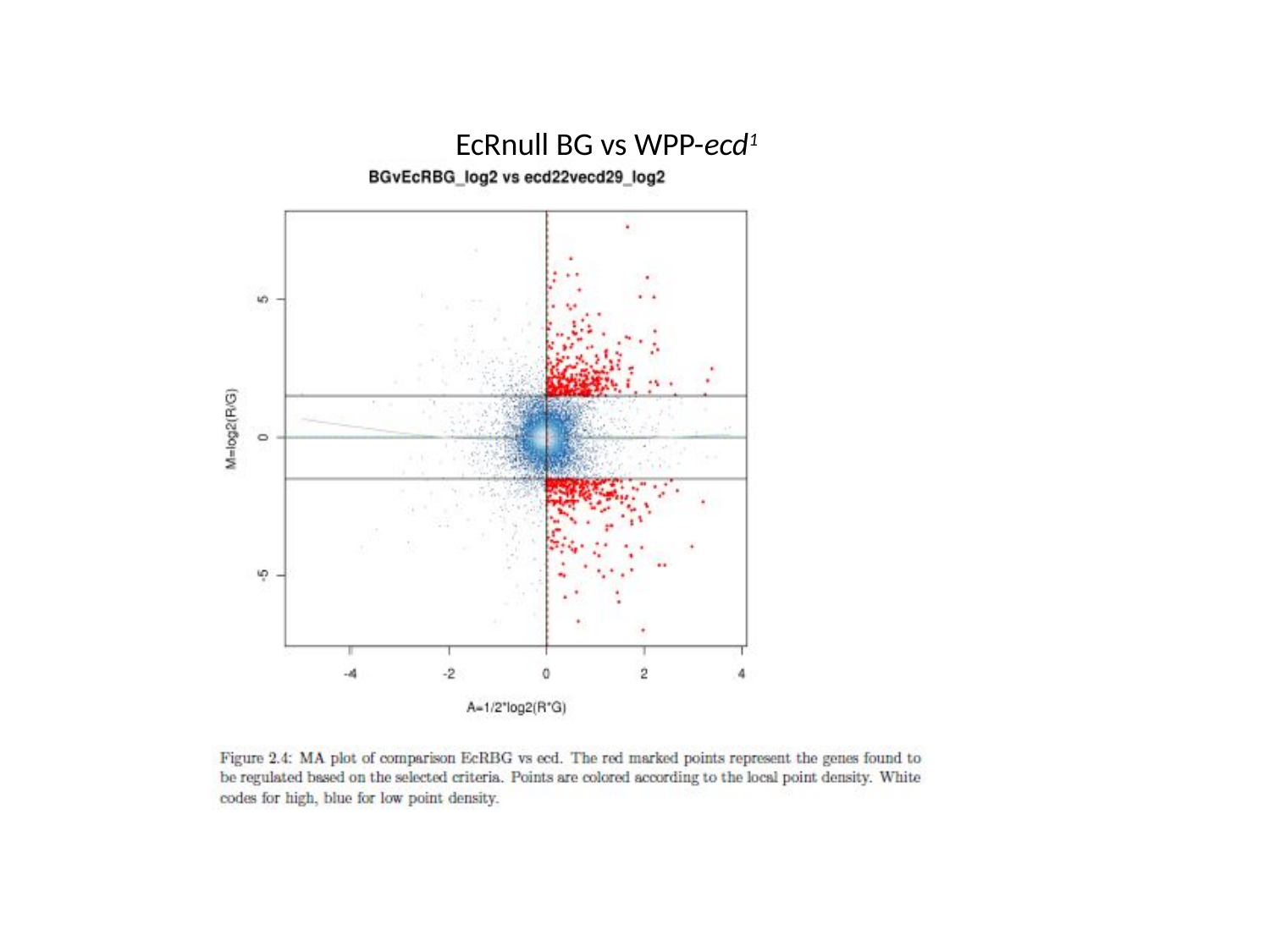

EcRnull BG vs WPP-ecd1

## Slide 4
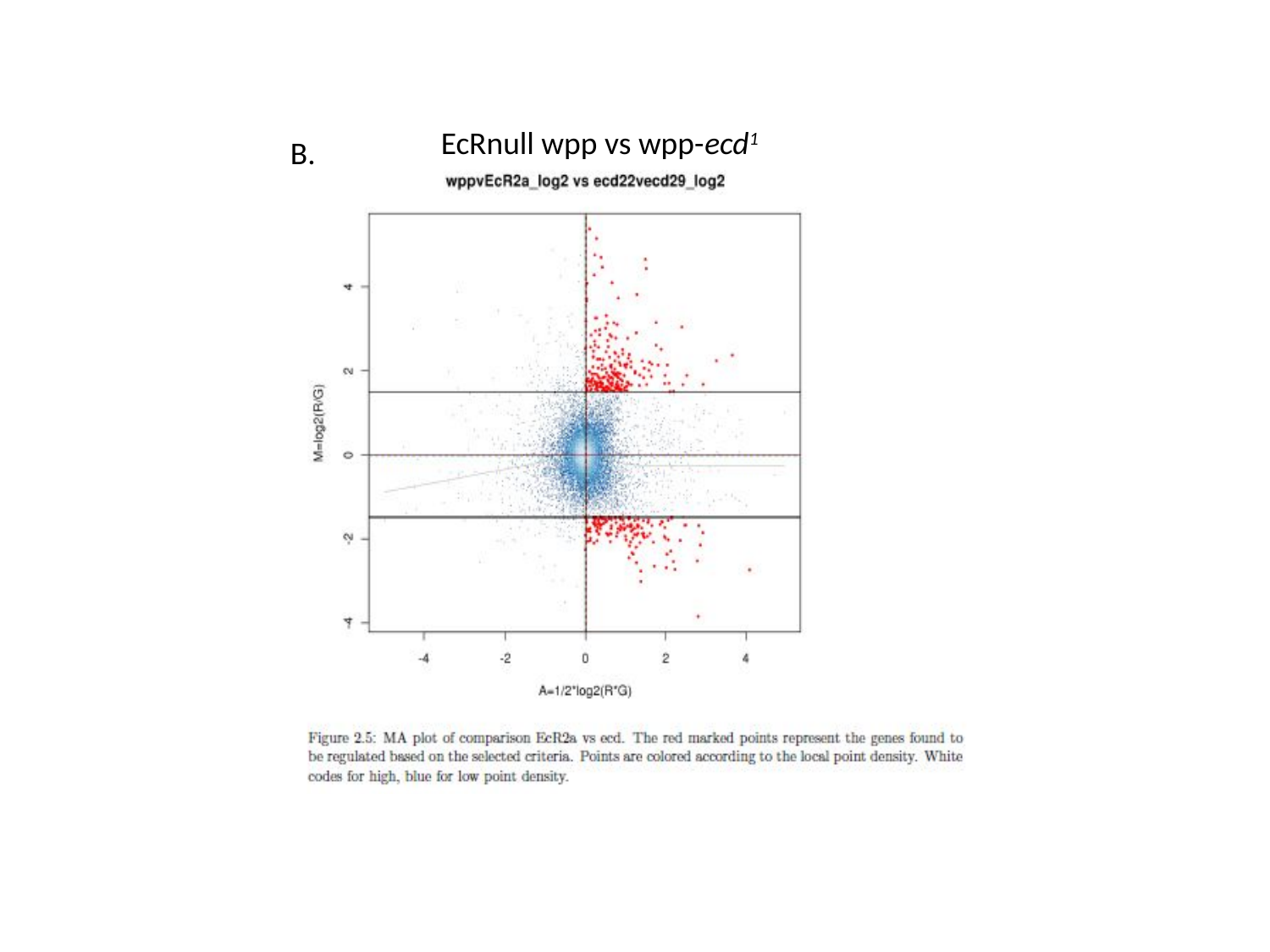

EcRnull wpp vs wpp-ecd1
B.

## Slide 5
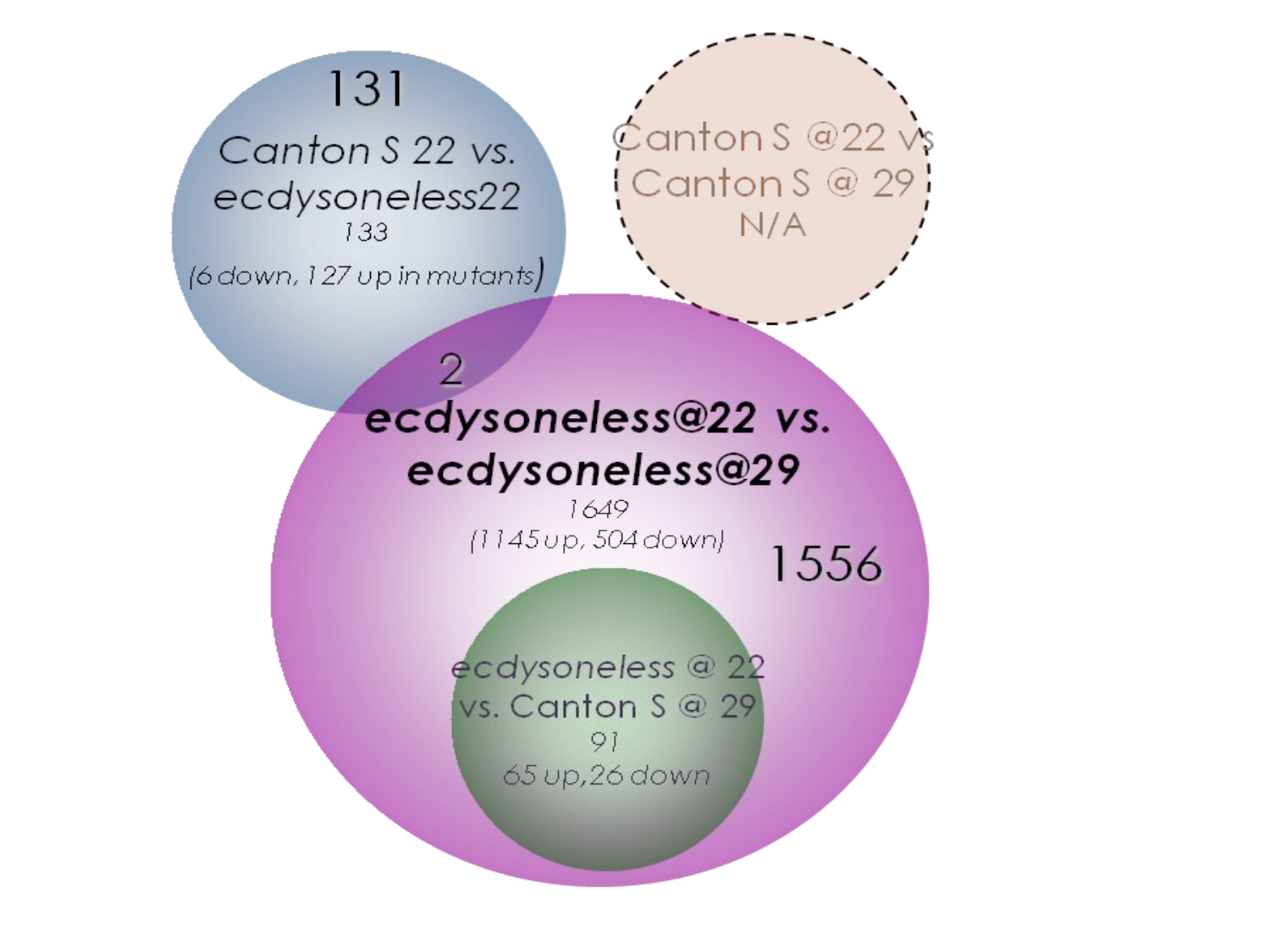

## Slide 6
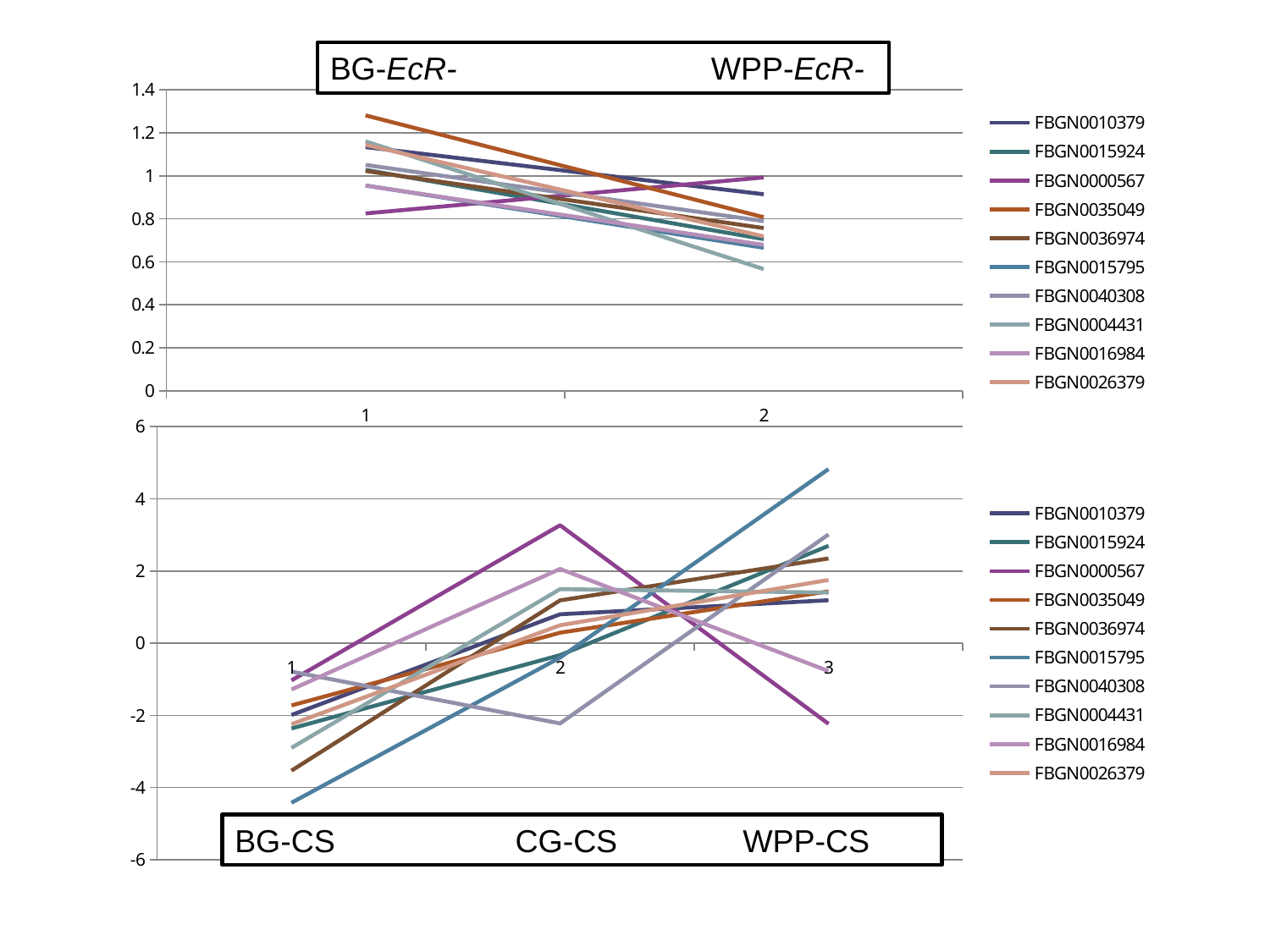

BG-EcR-		WPP-EcR-
### Chart
| Category | FBGN0010379 | FBGN0015924 | FBGN0000567 | FBGN0035049 | FBGN0036974 | FBGN0015795 | FBGN0040308 | FBGN0004431 | FBGN0016984 | FBGN0026379 |
|---|---|---|---|---|---|---|---|---|---|---|
### Chart
| Category | FBGN0010379 | FBGN0015924 | FBGN0000567 | FBGN0035049 | FBGN0036974 | FBGN0015795 | FBGN0040308 | FBGN0004431 | FBGN0016984 | FBGN0026379 |
|---|---|---|---|---|---|---|---|---|---|---|BG-CS		 CG-CS 	WPP-CS

## Slide 7
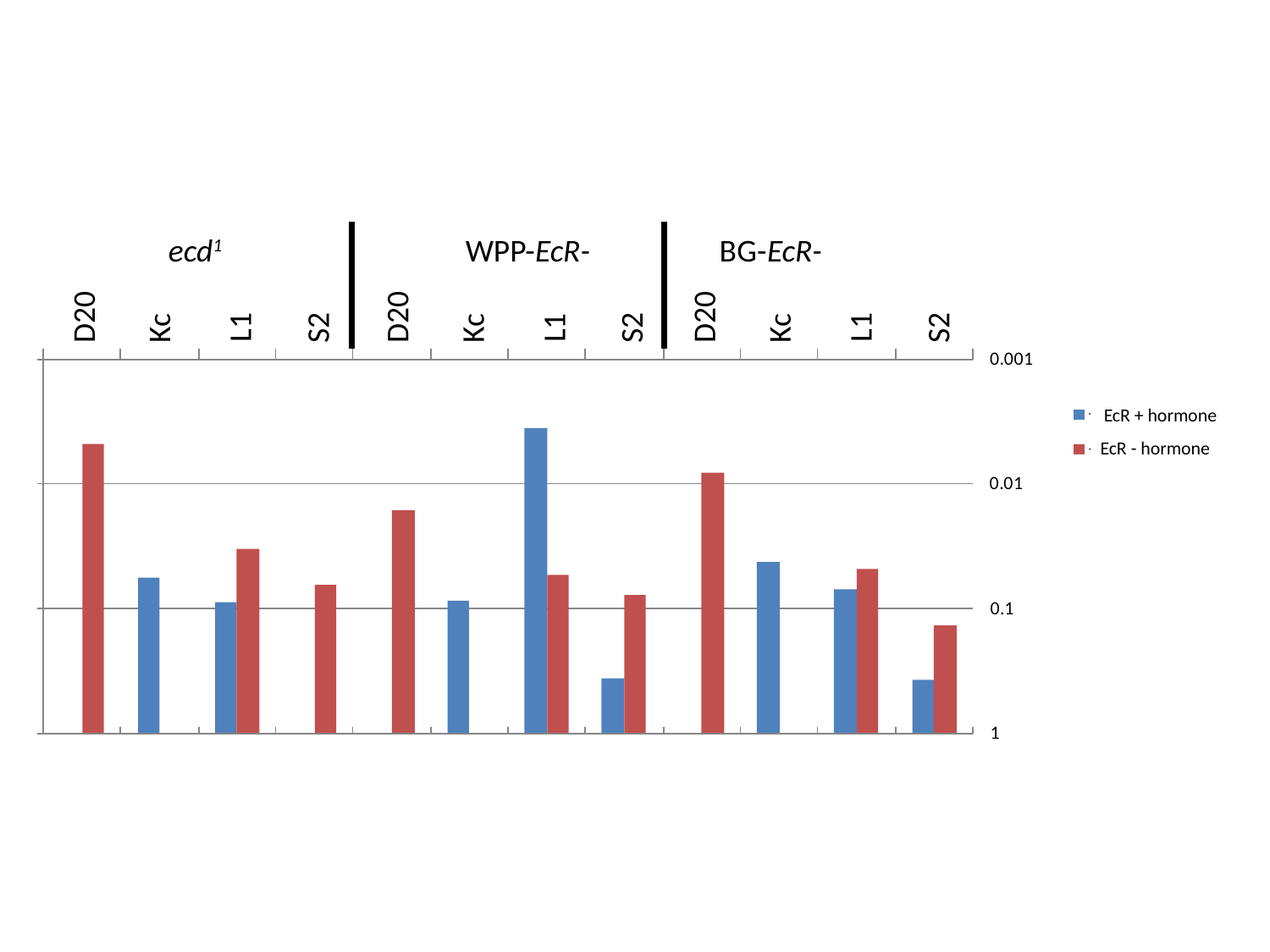

ecd1 	 WPP-EcR- BG-EcR-
D20
D20
D20
L1
S2
S2
L1
S2
L1
Kc
Kc
Kc
EcR + hormone
EcR - hormone
